# *In-silico* study of biotic and abiotic stress-related transcription factor binding sites in the promoter regions of rice *germin-like protein* genes

**DOI:** 10.1371/journal.pone.0211887

**Published:** 2019-02-14

**Authors:** Arpita Das, Krishnendu Pramanik, Rishu Sharma, Saikat Gantait, Joydeep Banerjee

**Affiliations:** 1 Genetics and Plant Breeding, RRS-Chakdah, Bidhan Chandra Krishi Viswavidyalaya, Mohanpur, Nadia, West Bengal, India; 2 Agricultural Biotechnology, Bidhan Chandra Krishi Viswavidyalaya, Mohanpur, Nadia, West Bengal, India; 3 Plant Pathology, Bidhan Chandra Krishi Viswavidyalaya, Mohanpur, Nadia, West Bengal, India; 4 Crop Research Unit, Directorate of Research, Bidhan Chandra Krishi Viswavidyalaya, Mohanpur, Nadia, West Bengal, India; 5 Department of Agricultural and Food Engineering, Indian Institute of Technology Kharagpur, Kharagpur, Paschim Medinipur, West Bengal, India; ICAR-Indian Institute of Agricultural Biotechnology, INDIA

## Abstract

Germin-like proteins (GLPs) are involved in biotic and abiotic stress tolerance in different plant species. Rice (*Oryza sativa* L.) genome contains about 40 GLP family member proteins in nine chromosomes. Although some of the rice *GLP* (*OsGLP*) promoters have been studied through *in silico* analysis as well as experimentally, studies regarding the distribution pattern of the biotic and abiotic stress associated transcription factor binding sites (TFbs) in the promoter regions of *OsGLP* genes have not been attempted thoroughly. Several transcription factors (TFs) namely NAC, WRKY, bHLH, bZIP, MYB and AP2/ERF act as major TFs concerned with biotic as well as abiotic stress responses across various plant species. In the present study the *in silico* analysis was carried out using the 1.5 kilobases (kb) promoter regions from 40 different *OsGLP* genes for the presence of NAC, WRKY, bHLH, bZIP, MYB and AP2/ERF TFbs in it. Among various *OsGLP* gene promoters, *OsGLP8-11* was found to contain highest number of tested TFbs in the promoter region whereas the promoter region of *OsGLP5-1* depicted least amount of TFbs. Phylogenetic study of promoter regions of different *OsGLP* genes revealed four different clades. Our analyses could reveal the evolutionary significance of different *OsGLP* gene promoters. It can be presumed from the present findings as well as previous reports that *OsGLP* gene duplications and subsequent variations in the TFbs in *OsGLP* gene promoter regions might be the consequences of neofunctionalization of *OsGLP* genes and their promoters for biotic and abiotic stress tolerance in rice.

## Introduction

Germin-like proteins (GLP) belonging to cupin superfamily are evolutionary conserved plant glycoproteins [1]. Germin protein was reported for the first time in germinating wheat grain and it was thought to be a marker gene for germination [2]. Later on GLP proteins have been identified in other angiosperms, gymnosperms as well as mosses [3] and their nomenclature have been given due to their structural similarity with germin proteins as well as their presence in the same superfamily like germin. Further studies established that GLP proteins possess unique biochemical as well as physiological functions across plant species [1]. Majority of the GLP family members have been involved in biotic as well as abiotic stress responses [1, 3–5] in diverse plant.

Rice (*Oryza sativa* L.) is used as a major staple food for more than half of the world’s population. Since the availability of genomic sequence information in public database, several studies have been conducted to characterize individual GLP family members from rice and other crops [1]. An interesting study on rice reported 12 *OsGLP* gene families as quantitative traits loci (QTL) associated with blast disease and all of those genes were present on chromosome number 8 [6]. Additionally, RNAi-mediated gene silencing of rice germin-like protein1 (*OsGLP1*) depicted susceptibility of *OsGLP1*-down regulated plants to sheath blight and blast diseases of rice compared to untransformed control plants [7]. Further study in a heterologous system of tobacco revealed that over-expression of *OsGLP1* gene improved resistance to *Fusarium solani* infestation in the transgenic tobacco lines [8]. In addition to biotic stresses several *GLP* genes have been found to be involved in abiotic stress mechanisms also. RNAi-mediated down-regulation of *OsGLP1* has documented improved salt tolerance of transgenic lines during seed germination and early seedling growth compared to untransformed plant [3]. Another study has identified the expressional changes of 31 different *OsGLP* genes upon exposure to drought, salt, cold and heavy metal stresses [9].

In association with different *GLP* genes, their promoters have also been found to be involved in biotic and abiotic stress responsiveness. A rice *GLP* gene (*OsRGLP*2) promoter showed wound inducible property as well as abiotic stress (dehydration and salt stress) responsiveness [10]. Another detailed study on transgenic potato plants harboring promoter::*GUS* fusion construct containing 1107 base pair (bp) promoter region of *OsRGLP2*, depicted expressional up-regulation of GUS fusion protein in response to two fungal pathogens namely *F*. *solani* and *Alternaria solani* [11]. Study has been conducted to analyze the *OsGLP1* and a putative germin A promoter region from five Pakistani rice varieties and it revealed that the TATA box binding protein could also recognize regions other than the (-30) TATA box element on the promoter sequences [12]. In addition to rice, the *GLP* gene promoters have also been characterized in other crops. In an earlier study, a *GLP* gene (*PcGER1*) promoter was cloned from *Pinus caribaea* and the 1520 bp upstream promoter region was characterized in tobacco Bright Yellow 2 (BY‐2) cells upon exposure to different phytohormones [13]. Maximum promoter activity was detected in day 4 upon exposure to 2,4-D and BA [13]. In barley (*Hordeum vulgare*), eight different germin-like protein (*GER4*) gene promoters have been analyzed and it has been found that different promoters showed diverse response upon pathogen attack preferably due to the presence of W-box domain in the TATA-box proximal promoter region [14]. Another study on barley at evolutionary point of view suggested that in case of barley *GER4* gene, the promoter region rather than the coding DNA sequence (CDS) region have been diversified after duplication of the genomic region to subsequently acquire new function [4].

The adaptive strategy of plants under biotic and abiotic stress conditions include expression as well as utilization of several transcription factors (TFs) which eventually regulate a number of pathogenesis related genes (*PR* genes) or signaling genes after binding with their promoter regions [15–16]. The adequate expression of a gene under a particular promoter is governed by the presence of appropriate transcription factors binding sites (TFbs) in the promoter region. Some major TFs like NAM/ATAF1/CUC2 (NAC), N-terminal WRKY domain containing TF (WRKY), basic helix-loop-helix (bHLH), basic leucine zipper (bZIP), myeloblastosis viral oncogene homolog TF (MYB) and APETALA 2/ethylene-responsive element binding factor (AP2/ERF) are very well documented in plants for their abiotic and biotic stress responses. In addition to these, TFs like C2H2, Zinc finger protein and MADS box are also having well established role in plant immunity [17–19].

Although, a large number of GLPs are available in rice, their exact number and proper nomenclature have been found to be highly confusing [9, 20]. A recent study documented the existence of 43 *OsGLP* members from rice and that particular study revealed expressional analysis of different *OsGLP* members upon exposure to various abiotic stresses at differential developmental stages [9]. Beside the *in silico* promoter analysis of two rice *GLP* promoters namely *OsRGLP1* and *OsRGLP2*; functional characterizations of some *OsGLP* gene promoters have also been conducted [10–11, 21–22]. It is presumed that the promoter regions of these multigene family members play crucial role regarding the concerned gene expression under differential biotic and abiotic stresses as detected in other crops [4, 14]. In the present *in silico* study, the upstream 1.5 kilobases (kb) region of 40 *OsGLP* gene promoters have been analyzed for putative availability of the major biotic and abiotic stress-related transcription factor binding sites (TFbs). Additionally, the phylogenetic study of *OsGLP* gene promoters, their evolutionary significance and the expression of *OsGLP* genes under respective gene promoters have also been interpreted here.

## Materials and methods

### Data retrieval of rice GLP sequences

Protein sequences of *OsGLP* genes were collected from Rice Genome Annotation Project Database (https://rapdb.dna.affrc.go.jp/) and from National Center for Biotechnology Information database (https://www.ncbi.nlm.nih.gov/). All of the mRNA sequences for each *OsGLP* were retrieved from NCBI database. The coding DNA sequences (CDS) of each *OsGLP* were blasted at NCBI using Ref Seq Representative Genome database and their corresponding DNA sequence was identified from different rice chromosome. As the length of 5′ UTR (untranslated region) of different *OsGLP* mRNA were found to differ in length [9], the upstream 1500 bp region from CDS were treated as promoter region for uniformity. The region analyzed as promoter was comprised of 5′ UTR (if any) along with the core promoter region as well as distal regulatory elements. All sequences were based on *O*. *sativa* Japonica Group cultivar Nipponbare.

### Phylogenetic analysis and chromosomal organization

Phylogenetic analysis of 40 different promoter regions of *OsGLP* genes (i.e. 1.5 kb upstream regulatory region from CDS) was conducted through Neighbor-Joining method using Molecular Evolution Genetic Analysis 7 (MEGA 7) software [23]. The percentage of replicate trees in which the associated taxa clustered together in the bootstrap test (1000 replicates) has been shown next to the branches. Construction of Models with the lowest BIC scores (Bayesian Information Criterion) were considered to describe the substitution pattern in a comprehensive manner. The BIC value was 82388.402 and the AICc (Akaike Information Criterion) value was 81718.828 involving 40 nucleotide sequences of 1.5 kb *OsGLP* gene promoters. All positions containing gaps and missing data were eliminated during evaluation. There were a total of 801 positions in the final dataset. Tajima’s neutrality test was conducted using the above mentioned software for finding out nucleotide diversity. During selection of the model, nucleotide frequencies and rates of base substitutions for each nucleotide pair were considered in MEGA 7. Chromosomal organization of 40 *OsGLP* genes were performed with the help of Rice Database Oryzabase-Shigen (https://shigen.nig.ac.jp/rice/oryzabase/) using the Chromosome Map Tool (http://viewer.shigen.info/oryzavw/maptool/MapTool.do). Different *OsGLP* genes which were separated by a maximum of five genes were designated as duplicated genes in accordance to published protocol [9]. The presence of *OsGLP* genes on positive or negative strand of the DNA, were counted for considering them as tandemly or inverse tandemly duplicated genes, respectively.

### Analysis of the TFbs

Promoter regions of 40 *OsGLP* genes were analyzed using PlantPAN 2.0 software (http://plantpan2.itps.ncku.edu.tw/) for identification of transcription factor binding sites (TFbs) in *OsGLP* gene promoters. Multiple promoter analysis programme (http://plantpan2.itps.ncku.edu.tw/gene_group.php?#multipromoters) was used and occurrence of same TFbs in different promoter regions were identified. TFbs for different TFs involved in biotic and abiotic stresses namely NAC, WRKY, bHLH, bZIP, MYB and AP2/ERF were studied in 40 *OsGLP* gene promoters. A total of 1.5 kb upstream sequence was analyzed for each GLP member and it was divided into three regions namely proximal promoter region (500 bp upstream from the start codon), median promoter region (501 bp to 1000 bp upstream from the start codon) and distal promoter region (1001 bp to 1500 bp upstream from the start codon).

### Expression study of *OsGLP* genes

*OsGLP* gene expression data was extracted by GENEVESTIGATOR from *O*. *sativa* database using Affymatrix Rice Genome Array platform (OS_AFFY_RICE-0) and ‘Perturbations’ tool was used to find out the differential gene expression under biotic and abiotic stresses. The fold change in gene expression was detected using filter 2.0 fold as benchmark. The fold changes in the expression of *OsGLP* genes under different biotic and abiotic stress conditions (S2 and S3 Files) were used to generate gene expression heatmap using Heatmapper online tool (http://www1.heatmapper.ca/expression/) using Red/Green colour scheme where “Red” colour shows down-regulation and “Green” colour shows up-regulation of respective genes. The microarray dataset used for *OsGLP* gene expression study using GENEVESTIGATOR has been presented in S2 and S3 Files.

## Results and discussion

### Chromosomal organization of *OsGLP* gene promoters

**Fig 1:**
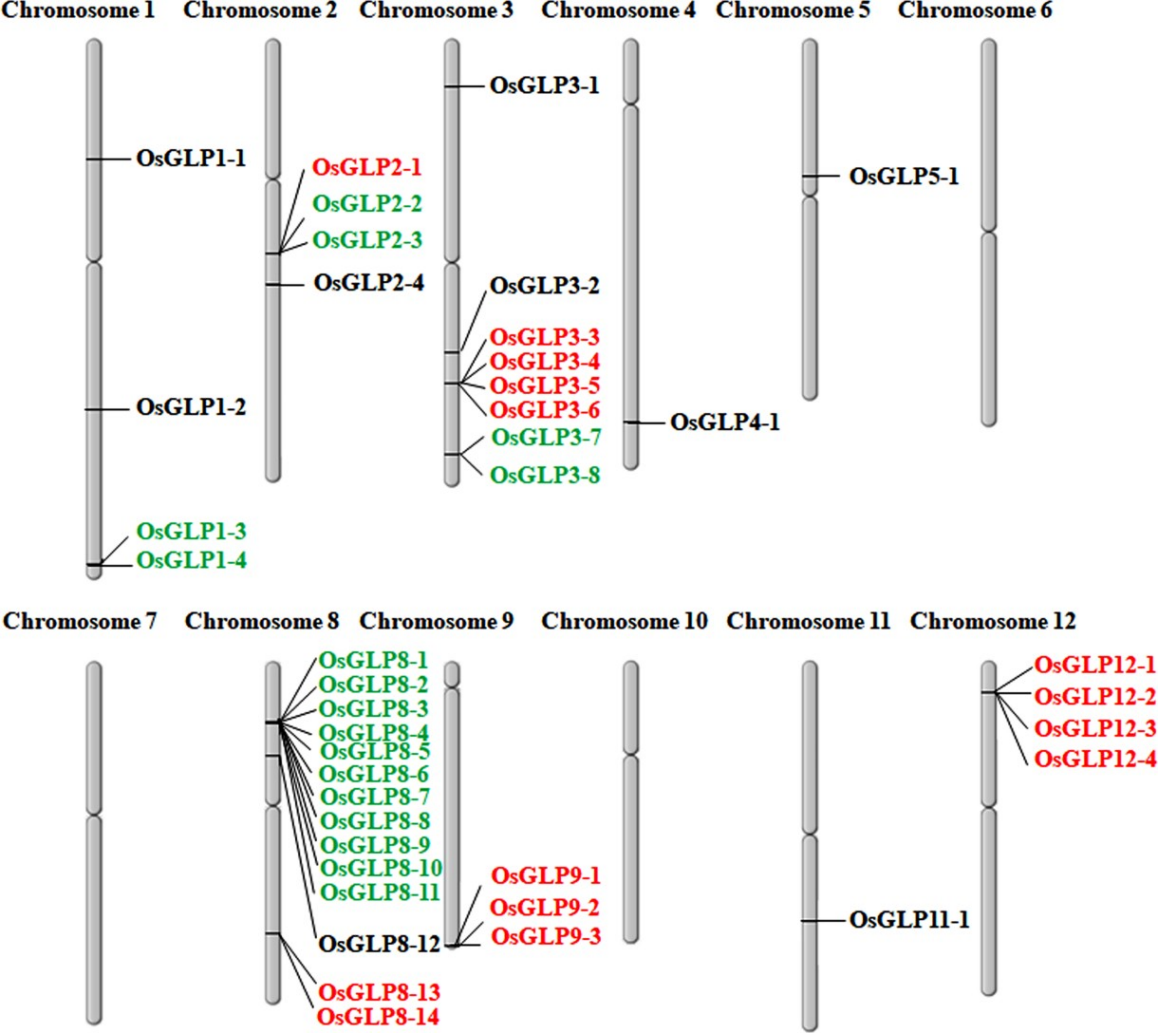
Chromosomal distribution of 40 different rice *GLP* (*OsGLP*) genes. Tandem and inverse tandem duplication of *OsGLP* on different chromosomes are shown in green and red color, respectively.

Characteristics of 40 *OsGLP* gene promoters of *O*. *sativa* retrieved from NCBI database have been depicted in Table 1 and their location on each chromosomes have also been presented (Fig 1). The name of the OsGLP protein, the alternate name of some of the genes coding OsGLP (given in parentheses), their corresponding protein ID (accession number), the promoter regions of each *OsGLP* genes studied here and chromosomal locus of the respective *OsGLP* gene has been described (Table 1 and S1 File).

**Table 1:** Description of unique rice *GLP* (*OsGLP*) genes and their promoter regions.

GLP name	Protein id	Promoter region	Locus on chromosome	Strand
***OsGLP1-1***	XP_015622834.1	10170426–10168927	Os01g0284500	- strand
***OsGLP1-2***	XP_015613392.1	29230400–29231899	Os01g0705100	+ strand
***OsGLP1-3***	XP_015644058.1	41913324–41914823	Os01g0952000	+ strand
***OsGLP1-4***	XP_015644512.1	41916366–41917865	Os01g0952100	+ strand
***OsGLP2-1***	XP_015624078.1	17167497–17165998	Os02g0491600	- strand
***OsGLP2-2***	XP_015624077.1	17165917–17167416	Os02g0491700	+ strand
***OsGLP2-3***	XP_015626751.1	17173722–17175221	Os02g0491800	+ strand
***OsGLP2-4***	XP_015622959.1	19599980–19598481	Os02g0532500	- strand
***OsGLP3-1***	XP_015633295.1	4156170–4157669	Os03g0179100	+ strand
***OsGLP3-2***	XP_015630224.1	25314197–25315696	Os03g0651800	+ strand
***OsGLP3-3***	XP_015629955.1	27781471–27779972	Os03g0693700	- strand
***OsGLP3-4***	XP_015630078.1	27786081–27784582	Os03g0693800	- strand
***OsGLP3-5***	XP_015629893.1	27790413–27788914	Os03g0693900	- strand
***OsGLP3-6***	XP_015629049.1	27793400–27791901	Os03g0694000	- strand
***OsGLP3-7***	XP_015628931.1	33584974–33586473	Os03g0804500	+ strand
***OsGLP3-8***	XP_015630140.1	33589695–33591194	Os03g0804700	+ strand
***OsGLP4-1***	XP_015634976.1	31396595–31395096	Os04g0617900	- strand
***OsGLP5-1***	XP_015638422.1	11465690–11467189	Os05g0277500	+ strand
***OsGLP8-1***	XP_015649422.1	5185375–5186874	Os08g0188900	+ strand
***OsGLP8-2 (Germin-like protein 16)***	XP_015648581.1	5206934–5208433	Os08g0189100	+ strand
***OsGLP8-3***	XP_015650201.1	5220775–5222274	Os08g0189200	+ strand
***OsGLP8-4 (OsGer1)***	XP_015650202.1	5227380–5228879	Os08g0189300	+strand
***OsGLP8-5***	XP_015650206.1	5232364–5233863	Os08g0189400	+ strand
***OsGLP8-6***	XP_015650204.1	5237572–5239071	Os08g0189500	+ strand
***OsGLP8-7***	XP_015650203.1	5241098–5242597	Os08g0189600	+ strand
***OsGLP8-8***	XP_015648995.1	5247290–5248789	Os08g0189700	+ strand
***OsGLP8-9***	XP_015648901.1	5252864–5254363	Os08g0189850	+ strand
***OsGLP8-10 (OsRGLP2)***	XP_015651068.1	5258764–5260263	Os08g0189900	+ strand
***OsGLP8-11 (OsRGLP1)***	XP_015651069.1	5262817–5264316	Os08g0190100	+ strand
***OsGLP8-12***	XP_015648080.1	7997132–7995633	Os08g0231400	- strand
***OsGLP8-13***	XP_015649499.1	22555500–22554001	Os08g0459700	- strand
***OsGLP8-14 (OsGer5*,** ***OsGLP1)***	XP_015648639.1	22560443–22558944	Os08g0460000	- strand
***OsGLP9-1***	XP_015611452.1	22697583–22696084	Os09g0568500	- strand
***OsGLP9-2***	XP_015612249.1	22699889–22698390	Os09g0568600	- strand
***OsGLP9-3***	XP_015612248.1	22702365–22700866	Os09g0568700	- strand
***OsGLP11-1***	XP_015618049.1	19584185–19585684	Os11g0537350	+ strand
***OsGLP12-1***	XP_015619653.1	2691258–2689759	Os12g0154700	- strand
***OsGLP12-2***	XP_015619655.1	2694943–2693444	Os12g0154800	- strand
***OsGLP12-3***	XP_015619657.1	2698714–2697215	Os12g0154900	- strand
***OsGLP12-4***	XP_015619658.1	2701472–2699973	Os12g0155000	- strand

Out of these 40 *OsGLP* genes, chromosome 1 and 2 were found to possess four *OsGLP* genes in each and 8 *OsGLP* genes were observed to be located in chromosome 3 (Fig 1). Chromosome 4, 5 and 11 were detected with single *OsGLP* gene in each while chromosome 8 was found to possess highest number of *OsGLP* genes (14) and 3 *OsGLP* genes were identified in chromosome 9 (Fig 1). Although a recent study documented 43 different *OsGLP* genes from rice [9], the promoter regions of only 40 *OsGLP* members have been characterized here. The CDS sequence of *OsGLP5-2* belonging to locus LOC_Os05g19670 [9] showed 100% homology with *Oryza sativa japonica* Group *OsGLP5-1* (accession number XM_015782936). Additionally, the protein sequence of *OsGLP1-5* with locus number LOC_Os01g72300 [9] was used to blast at NCBI protein database and it depicted 100% homology with OsGLP1-4 protein sequence. Nevertheless, another *GLP* (*OsGLP3-9*) belonging to the locus LOC_Os03g58990 mentioned in earlier report [9] depicted maximum homology with a hypothetical protein. Consequently, the CDS of *OsGLP3-9* showed maximal homology with CDS sequence of *OsGLP3-8* gene of *Oryza sativa japonica* group and no other mRNA from japonica group exhibited significant homology with that *GLP*. Hence, only 40 unique *OsGLP* gene promoters have been considered here. These large numbers of *OsGLP* gene promoters might have been generated following the destinies of duplicated *OsGLP* genes through neofunctionalization of the promoter region as revealed in barley *GLP* gene cluster [4, 14]. Due to the presence of related sequences in close proximity and their availability in both the DNA strands on chromosome 8, it was speculated that eleven of the *OsGLP (OsGLP8-1*, *-2*, *-3*, *-4*, *-5*, *-6*, *-7*, *-8*, *-9*, *-10 and -11)* genes exhibited tandem duplications while *OsGLP8-13* and *OsGLP8-14* exhibited inverse tandem duplications (Fig 1 and Table 1). Additionally, *GLP* gene cluster were available as both tandem and inverse tandem duplications in chromosome number 2 and 3 whereas, in chromosome number 12 and 9, only inverse tandem duplications were prevailed. On the other hand, in chromosome 1 only tandem duplication was revealed (Fig 1). An earlier study highlighted the significance of tandem duplications in *OsGLP* gene families [9], however, specific reports of inverse tandem duplication was not discussed. During the course of evolution the multigene families have been developed through genome wide duplication, deletion, as well as translocation [24]. Several studies revealed that some tandemly duplicated genes in human as well as other genomes are expressed in opposite orientation from its nearby duplicated genes [25]. Mutation study in *Salmonella enterica* revealed that tandem inversion duplication may arise in the population due to selection pressure [26] and such type of duplication is known as reverse tandem duplication or inverted tandem duplication [27]. Based on the direction of transcript expression *OsGLP* genes were classified as tandemly or inverse tandemly duplicated genes which were present in two different DNA strands. Gene duplication has evolutionary significance and tandem duplication especially in chromosome number 8 played major role in rice to withstand biotic and abiotic stresses [6, 10]. Earlier studies on abundance of duplicated genes and their novel roles in biotic and abiotic stress management resembled similar findings [14]. In addition to rice, a number of *GLP* genes have also been reported in other crops *viz*. *Arabidopsis*, peanut, tomato as well as barley [1] and these multigene family proteins have been involved in basal host resistance [28].

### Analysis of the TFs

In rice as well as other crop species the TF families (NAC, WRKY, bHLH, bZIP, MYB and AP2/ERF) have been categorized on the basis of their involvement in ABA-independent and ABA-dependent pathways [29]. In the following section the frequency and importance of studied transcription factors in the *OsGLP* gene promoter regions have been discussed elaborately considering their correlation with biotic and abiotic stress management.

#### NAC

**Fig 2:**
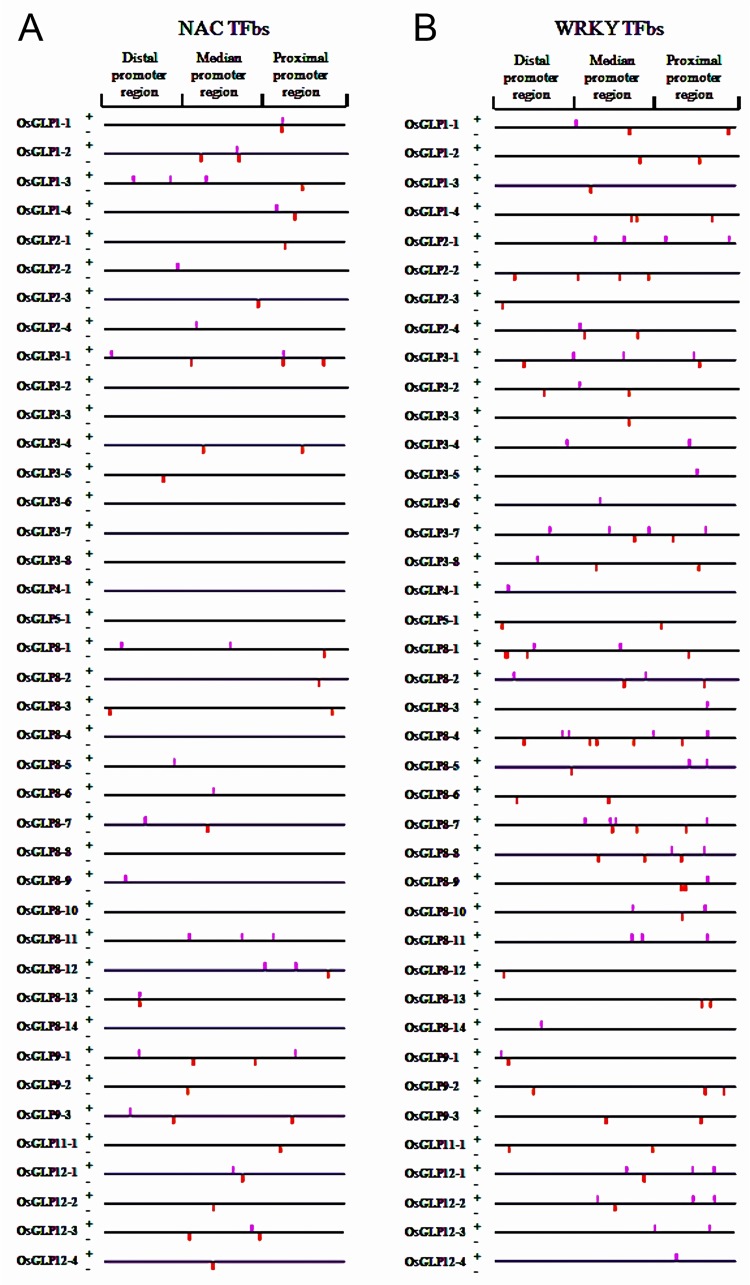
Distribution of NAC and WRKY transcription factor binding sites (TFbs) in 40 different *OsGLP* promoter regions. Putative NAC TFbs (A) and WRKY TFbs (B) located on positive and negative strands of DNA are shown as pink and red colored bars, respectively. The upstream 1.5 kilobases (kb) region of each *OsGLP* CDS were collected and analyzed as upstream regulatory region or promoter region. A total 1.5 kb upstream sequence was divided into three regions namely proximal promoter region (500 bp upstream from the start codon), median promoter region (501 bp to 1000 bp upstream from the start codon) and distal promoter region (1001 bp to 1500 bp upstream from the start codon).

A total of 58 NAC TFbs were recognized in 40 different *OsGLP* gene promoter regions (Fig 2A). The proximal promoter region was found to possess 20 NACbs in both the strands. The median region constituted of maximum number of NACbs (23) whereas relatively less number of NACbs (15) were observed in the distal promoter region. Bioinformatics analysis depicted that in the present study NAC TFs were found to recognize CATGTG sequence on DNA which were in accordance with the earlier literature [30]. In our study it was disclosed that in some of the *OsGLP* gene promoters (*OsGLP3-2*, *OsGLP3-3*, *OsGLP3-6*, *OsGLP3-7*, *OsGLP3-8*, *OsGLP4-1*, *OsGLP5-1*, *OsGLP8-4*, *OsGLP8-8*, *OsGLP8-10* and *OsGLP8-14*) NACbs were absent (S1 Table). Maximum number of NACbs was detected in *OsGLP3-1* which contained a total of 5 numbers of NACbs distributed throughout the regions. NAC TFs play major role in biotic and abiotic stress tolerance mechanism in plants and most of the *NAC* genes are induced by various abiotic stress signals [31]. Expression study in rice through microarray depicts that around 45 *NAC* genes are up-regulated upon exposure to abiotic stresses while more than 26 *NAC* genes show up-regulation in response to biotic stresses [32]. Additionally, in that study almost six different *NAC* genes depict differential expression in response to rice stripe virus and rice tungro spherical virus infestation. Over-expression of a *NAC* gene has been found to enhance drought resistance in transgenic rice at the reproductive stage, and also improve drought as well as salt tolerance in the vegetative stage [33]. In another study *OsNAC5* has been over-expressed in transgenic rice under root-specific promoter (*RCc3*), which depicted significantly higher yield compared to the non-transgenic lines under drought stress condition [34]. Similarly in another experiment, over-expression of *OsNAC9* under root-specific *RCc3* promoter exhibited increased grain yield in exposure to drought stress [35]. According to certain reports, over-expression of *NAC* in rice also improved tolerance to dehydration and high salinity, although with growth retardation and low reproductive yields [30, 36].

#### WRKY

In the present study, a total of 118 WRKY TFbs were detected covering all the *OsGLP* gene promoter regions (Fig 2B). The proximal promoter region was found to possess 43 WRKYbs in both the strands. The median region detected with having highest number of bs (49), whereas relatively lesser number of WRKYbs (26) were present in the distal promoter region. Interestingly, in case of *OsGLP* promoters in chromosome number 1, all the four members were devoid of any WRKYbs in the distal region (Fig 2B). However in case of *OsGLP* promoters in chromosome number 2 all the members lacked WRKYbs in the proximal region except *OsGLP2-1*, where not a single WRKYbs were found in the distal region of the promoter. *OsGLP3-6* promoter was the single one to have WRKYbs only in the median region. Among the rice *OsGLP* gene promoters the highest number of WRKYbs was found in the promoter region of *OsGLP8-4* followed by the promoter region of *OsGLP8-7* with the availability of 9 and 8 bs, respectively (S1 Table). WRKY is a conserve class of plant specific transcription factors responsible for defense and abiotic stress management in rice [37]. In the present study, WRKY TFs were expected to be recognized by TTGACN sequence in *OsGLP* gene promoters where N stands for any DNA bases. Generally the WRKY TFs recognize and attach with the W box ([T][T]TGAC[C/T]) of target gene promoters to modulate transcription [38], but according to certain reports WRKY TFs may bind to TTGACA [39] as well as TTGACG [40] motifs in promoter region. In rice, the role of WRKY as pathogenicity related systemic acquired resistance signaling transcription factors are well established [40]. Moreover, OsWRKY03 regulates cascade of signaling pathways mediated by jasmonic acid and salicylic acid for protecting plants from bacterial and fungal infections [41]. Along with the biotic stress involvement, a number of WRKY family members are also associated with abiotic stress responses in rice [38, 42]. The upregulation of some members of WRKY TFs have been found to implicate salt tolerance, drought tolerance and heat tolerance in plants [43, 44].

#### bHLH

**Fig 3:**
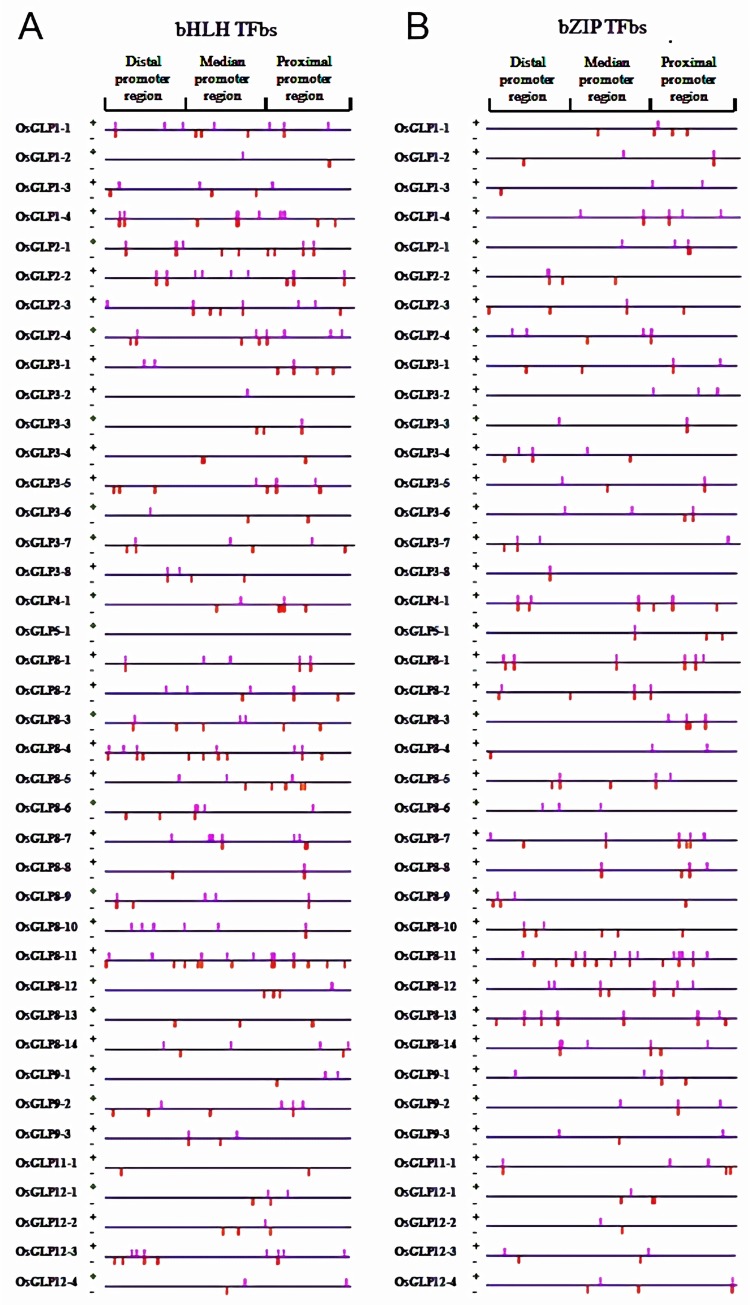
Distribution of bHLH and bZIP transcription factor binding sites (TFbs) in 40 different *OsGLP* promoter regions. Putative bHLH TFbs (A) and bZIP TFbs (B) located on positive and negative strands of DNA are shown as pink and red colored bars, respectively. The upstream 1.5 kilobases (kb) region of each *OsGLP* CDS were collected and analyzed as upstream regulatory region or promoter region. A total 1.5 kb upstream sequence was divided into three regions namely proximal promoter region (500 bp upstream from the start codon), median promoter region (501 bp to 1000 bp upstream from the start codon) and distal promoter region (1001 bp to 1500 bp upstream from the start codon).

In the present study a total of 292 bHLH TFbs were recognized (Fig 3A). The proximal, median and distal regions of *OsGLP* promoters were detected with 107, 92 and 93 bHLHbs, respectively in both the strands. Among the *OsGLP* gene promoters the highest number of bHLHbs was found in *OsGLP8-11* having 20 bs, while *OsGLP5-1* was devoid of any bHLHbs (S1 Table). Present study revealed several bHLHbs in *OsGLP* gene promoters and some of their consensus sequences were ATANN[A/T], NNNCG and [C/A][A/G]TATN. Earlier study revealed the binding of bHLH with canonical E-box sequences (CANNTG) for regulating gene expression [45]. Although some of the *OsGLP* gene promoters like *OsGLP1-4*, *OsGLP3-5*, *OsGLP3-6* and *OsGLP8-13* were found to possess “CANNTG” bs, most of them were found to have “CANNTN” sequence and it was recognized as probable bHLHbs. Previous findings confirmed the role of bHLH in inducing ABA-dependent signaling for management of cold stress [46]. Moreover, the involvement of bHLH in influencing the salicylic acid and jasmonic acid biosynthesis genes for conferring defence response has also been discussed in crop plants [47]. Additionally, bHLH TFs are involved in different abiotic responses as well as reactive oxygen species scavenging mechanisms [48]. In rice, some members of bHLH have been identified as an essential regulator of iron uptake and utilization [49] whereas; some other members of this TF family have been associated with regulating anthocyanin and anthocyanidine biosynthesis [50].

#### bZIP

It was found that a total of 232 bZIP TFbs were detected to be distributed throughout the studied promoter regions covering both the strands (Fig 3B). Most of the bZIPbs were located in the proximal region with relatively highest number of bs (upto 42%) followed by the median region revealing around 30% of bZIPbs availability. Among 40 *OsGLP* gene promoters, the highest number of bZIP specific bs (21) was observed in *OsGLP8-11* promoter, while the least bZIPbs (2 in each) were found in *OsGLP1-3*, *OsGLP3-8* and *OsGLP12-2* gene promoters (S1 Table). Earlier studies have depicted that plant bZIPs bind to the A-box (TACGTA), C-box (GACGTC) and G-box (CACGTG), but there are also reports of nonpalindromic binding sites for bZIPs [51]. In the present study, a number of A-box and G-box were detected in *OsGLP* gene promoters which might be acted as bZIPbs. In plants, bZIP transcription factors represent a divergent family of TFs which regulate several processes including light signaling, seed maturation, pathogen defence, flower development and abiotic stress signaling [51–52]. Extensive studies have confirmed the role of bZIP in ABA signaling in rice and it has been found to respond upon osmotic stress during vegetative growth [53]. Nevertheless, some bZIP have also been reported to induce salt stress resistance in *Arabidopsis* through interfering proteolysis and translocation from the endoplasmic reticulum to the nucleus and consequently up-regulation of salt stress genes [54]. Beside abiotic stress management, bZIP TFs regulate salicylic acid and *glutathione S-transferase 6* (*GST6*) gene signaling pathway and thus enable enhanced biotic stress tolerance in rice [55]. Earlier, it was observed that bZIP regulated accumulation of active oxygen species for facilitating programmed cell death and hypersensitive response [56].

#### MYB

**Fig 4:**
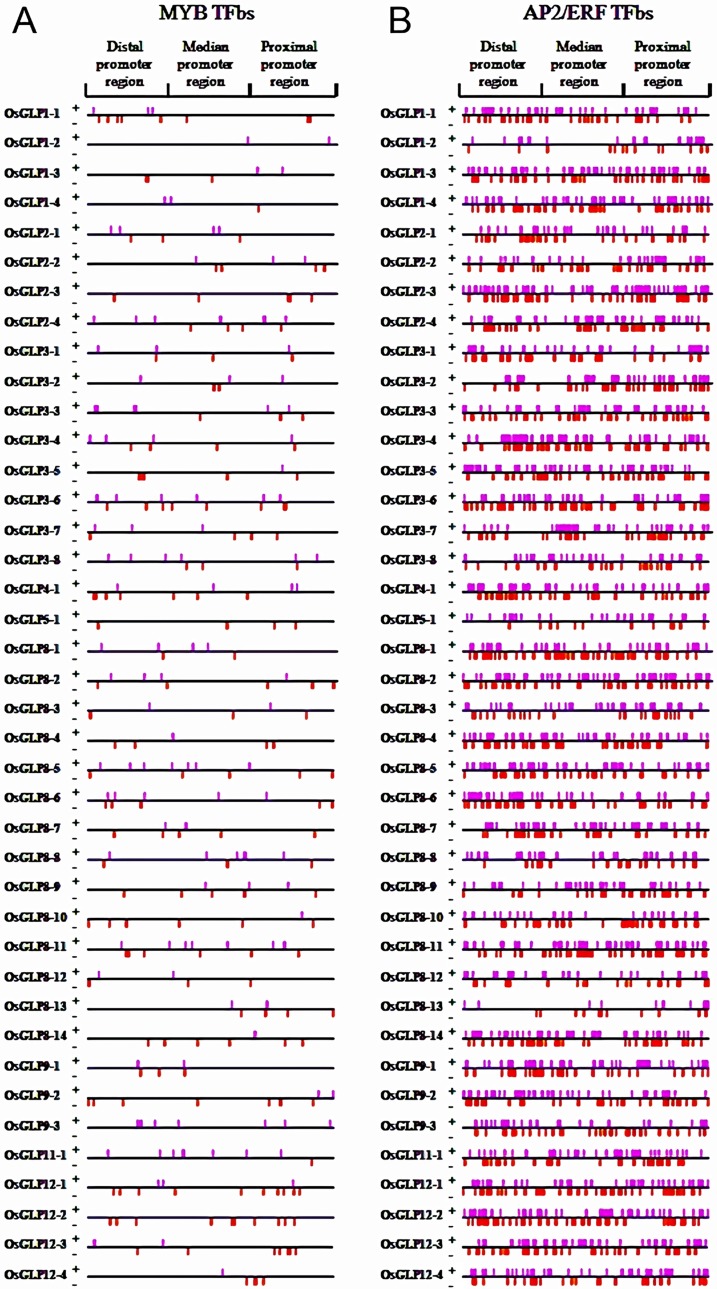
Distribution of MYB and AP2/ERF transcription factor binding sites (TFbs) in 40 different *OsGLP* promoter regions. Putative MYB TFbs (A) and AP2/ERF TFbs (B) located on positive and negative strands of DNA are shown as pink and red colored bars, respectively. The upstream 1.5 kilobases (kb) region of each *OsGLP* CDS were collected and analyzed as upstream regulatory region or promoter region. A total 1.5 kb upstream sequence was divided into three regions namely proximal promoter region (500 bp upstream from the start codon), median promoter region (501 bp to 1000 bp upstream from the start codon) and distal promoter region (1001 bp to 1500 bp upstream from the start codon).

A total of 308 MYB TFbs were recognized in 40 *OsGLP* gene promoter regions (Fig 4A). The distal portion had the highest number of bs (120) followed by the proximal region with 96 bs. The highest number of MYB TFbs was observed in *OsGLP3-6* followed by *OsGLP8-11* and *OsGLP12-1* containing 14, 13 and 13 bs, respectively (S1 Table). MYB members are considered as largest families of TFs in several crop plants. Previous studies indicated that consensus sequence like A(A/C)C(A/T)A(A/C)C, acted as MYB recognition elements (MREs) [57]. However, some previous report indicates WAACCA, TAACTG, CNGTTR, YAACKG, GGATA and CAACTG sequences as *cis*-acting regulatory elements for MYB binding in drought inducible gene expression [58]. In the present study it was revealed that MYB recognized GGATT and AGATT consensus sequences in the promoter regions of the studied *OsGLP* genes and that corroborated the earlier finding of plant MYB-related TF binding [59]. MYB members have been found to be involved in abiotic stress management in rice upon dehydration, salt and cold stresses [60]. Members of this TF also influenced ABA, PEG and SA-signaling pathways conferring response towards biotic and abiotic stresses [61]. Several functional genomics studies depict the involvement of MYBbs (G-box and H-box) in governing tissue specific expression and expressional up-regulation upon virus as well as bacterial infestation in plants [57, 62].

#### AP2/ERF

A total of 3238 bs were recognized in context to AP2/ERF TFs(Fig 4B). Grippingly all the AP2/ERFbs were found to be distributed throughout the promoter regions covering both positive and negative strands of proximal, median and distal portion. However, the distal portion was detected with highest number of AP2/ERFbs in comparison to other two portions. Interestingly, all of the studied *OsGLP* gene promoters had AP2/ERF binding sites. The highest number of AP2/ERFbs was observed in case of *OsGLP8-11* followed by *OsGLP12-3* comprised of 122 and 120 numbers of bs, respectively. *OsGLP8-13* exhibited least number of AP2/ERFbs (23) among all the 40 *OsGLP* gene promoters (S1 Table). Among the six TFs studied in the present context, the occurrence of AP2/ERFbs was the highest in the promoters of *OsGLP* gene families of rice. The relation of ERF family members with several stress responsive mechanism in plant species possibly increased its frequencies and distribution among stress-related gene promoters [29]. In the present study within the promoters of *OsGLP* gene families, AP2/ERF recognized several sequences as *cis*-acting elements (G/CTCTA, ATCTT/G/C, ATCAA etc.). In the earlier reports it was validated that ERF families could bind with two *cis*-acting elements *viz*. GCC box and CRT elements, involved in ethylene responsiveness related to *PR* genes and expression of cold and dehydration responsive genes, respectively [55]. An interesting review documented several binding sequences of AP2/ERFs [63] and some of those bs were available in *OsGLP* gene promoters. Several studies reported improved drought, salinity, water use efficiency, heat and cold response without compensating yield losses in transgenic rice plants through over-expression of *AP2/ERF* genes either in root-specific or in constitutive manner [64–65]. Additionally, some members of ERF families up-regulated the expression of gibberellin-deactivating gene under high-salinity stress causing reduction of the endogenous gibberellic acid (GA) level and consequently repressed growth as well as improved stress adaptation [66]. Regarding biotic stress response, AP2/ERFTF families induced the synthesis of ethylene, salicylic acid and jasmonic acid and increased the expression of *PR* genes after invasion by pathogen for enabling better resistance mechanism against pathogens [55]. It is worthy to mention that, according to certain reports up-regulation of genes by AP2/ERF members enhanced resistance to specific biotic and abiotic stresses and corrected growth defects [67–68].

**Fig 5:**
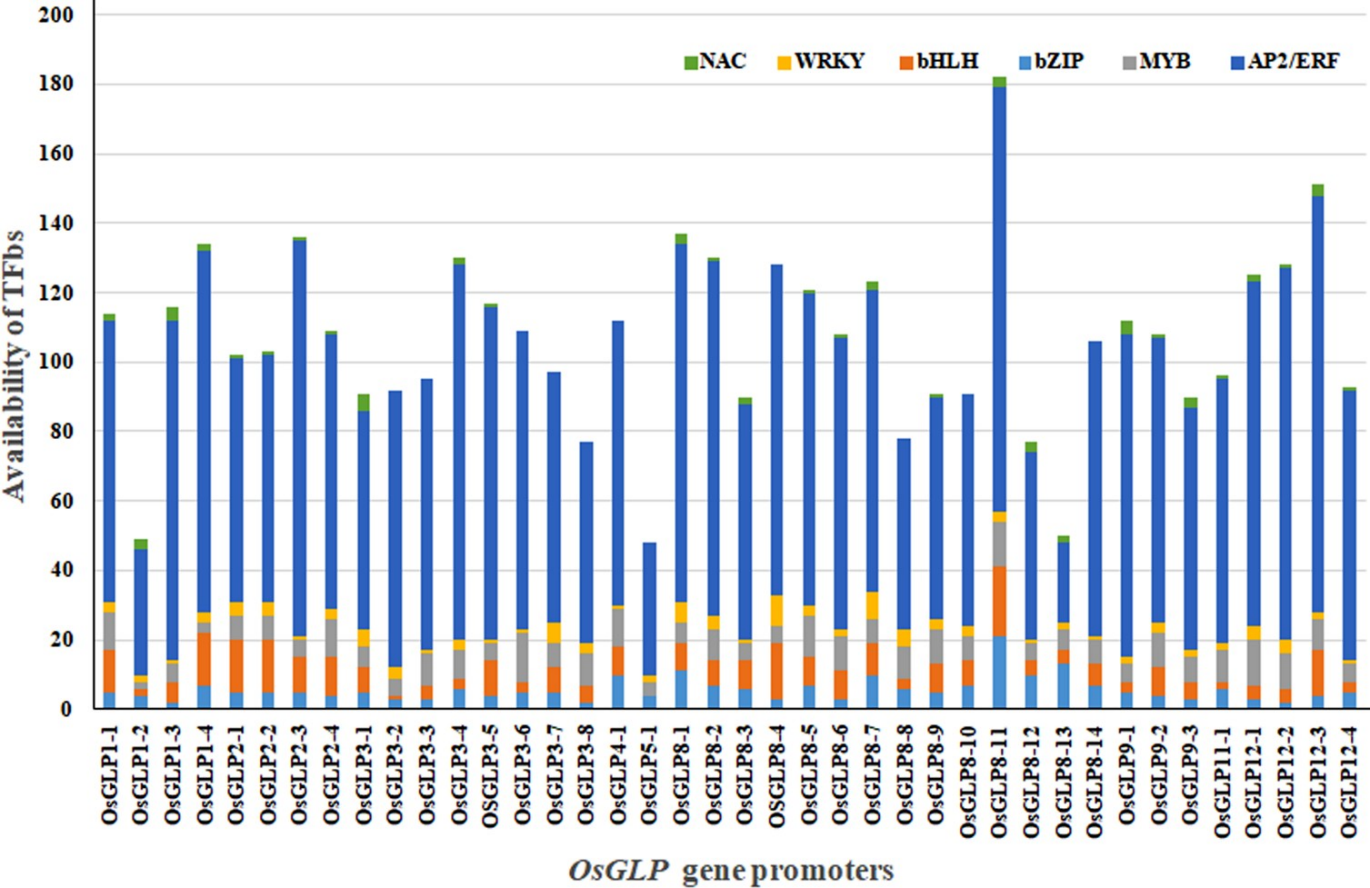
Relative availability of biotic and abiotic stress-related transcription factor binding sites (TFbs) in different *OsGLP* promoters. Number of TFbs for NAC, WRKY, bHLH, bZIP, MYB and AP2/ERF in different *OsGLP* promoters are shown in respective color.

In the present study on six TFbs availability among 40 *OsGLP* gene promoters, largest number of TFbs was detected in *OsGLP8-11* followed by *OsGLP12-3*, while least number of bs was identified in *OsGLP5-1* followed by *OsGLP1-2* (Fig 5 and S1 Table). Although, systematic analyses of most of the *OsGLP* gene promoters were not executed earlier in regards to biotic and abiotic stress-related TFbs availability, certain *in silico* studies were found to be in corroboration with our findings. Our analysis regarding locus identification revealed that *OsRGLP1* is similar to *OsGLP8-11* (Table 1), the highest TFbs possessing promoter. *In silico* promoter analysis by another group has demonstrated that the availability of a TFbs “ACGT” in *OsRGLP1* is far higher compared to *OsRGLP2* (equivalent to *OsGLP8-10*) promoter region [22]. According to earlier study, TFbs “ACGT” has been found to be involved in drought and senescence response [69] and it could be expected that *OsGLP8-11* might be highly responsive to drought stress. Further experimentation is needed with the high and low TFbs possessing *OsGLP* promoters to unravel their biological significance.

### Phylogenetic analysis of *OsGLP* gene promoters

**Fig 6:**
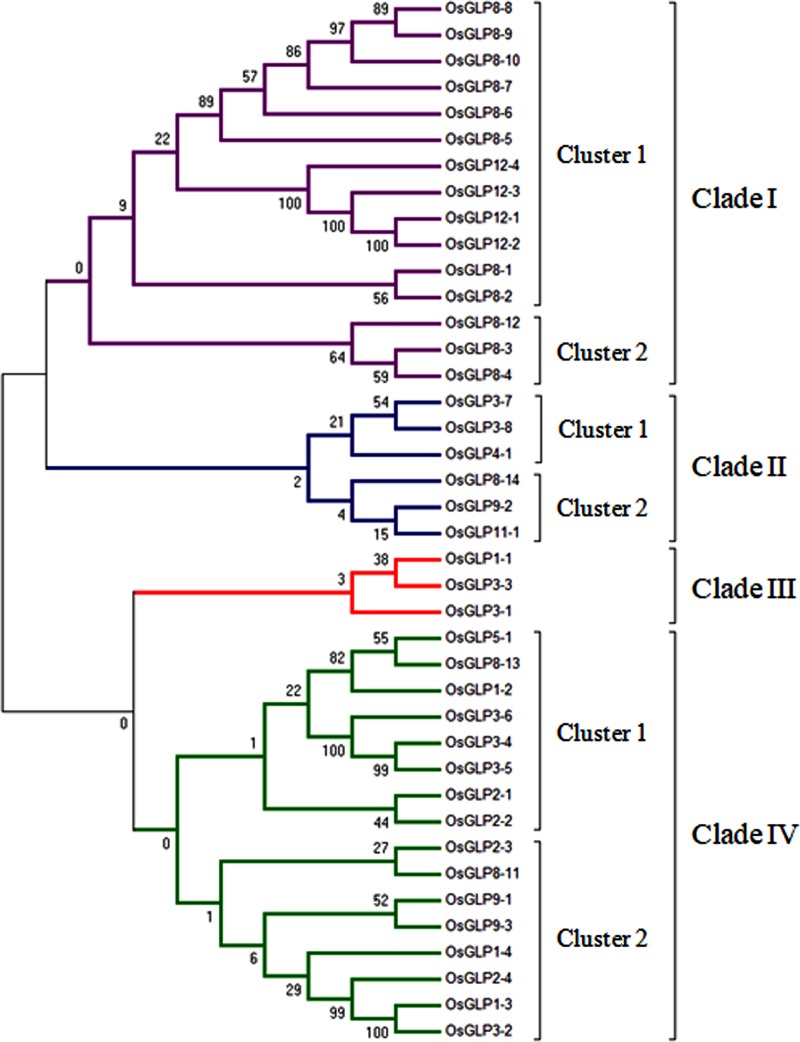
Relationship of 40 different *OsGLP* promoters. The phylogenetic tree is based on the 1.5 kilobases (kb) nucleotide sequence of the upstream region of each *OsGLP* CDS which has been analyzed as promoter regions of *OsGLP* mentioned in Table 1. Sequences were aligned using MEGA 7 and the evolutionary tree was constructed using the neighbor-joining method. Four different clades were identified in the phylogenetic analysis.

Phylogenetically, 40 *OsGLP* gene promoters were found to be divided into 4 different clades with bootstrap values of 0 to 100 (Fig 6). Clade I contained 15 sequences of which cluster 1 had 12 sequences (*OsGLP8-1*, *-2*, *-5*, *-6*, *-7*, *-8*, *-9*, *-10*, *OsGLP12-1*, *-2*, *-3* and *-4*) from chromosome 8 and 12, while the cluster 2 had 3 sequences (*OsGLP8-3*, *-4* and *-12*) belonging to chromosome 8. Clade II was comprised of 6 sequences having two clusters where, cluster 1 contained 3 sequences (*OsGLP3-7*, *-8* and *OsGLP4-1*) from chromosome 3 and 4 while the cluster 2 was composed of 3 promoter sequences (*OsGLP8-14*, *OsGLP9-2* and *OsGLP11-1*) from 3 different chromosomes (chromosome 8, 9 and 11). The clade III was revealed as the smallest clade and it consisted of only 3 sequences; of which two (*OsGLP3-1* and *OsGLP3-3*) were from chromosome 3 and one (*OsGLP1-1*) from chromosome 1. Clade IV was identified as the largest clade consisting of 16 promoter sequences of which 8 sequences (*OsGLP1-2*, *OsGLP2-1*, -*2*, *OsGLP3-4*, -*5*, -*6*, *OsGLP5-1* and *OsGLP8-13*) were in cluster 1 and remaining 8 sequences (*OsGLP1-3*, *-4*, *OsGLP2-3*, *-4*, *OsGLP3-2*, *OsGLP9-1*, *-3* and *OsGLP8-11*) were belonging to cluster 2. *OsGLP* promoters on the chromosome 12 showed maximum homology among themselves and *OsGLP* promoters on chromosome 8 also exhibited high level of similarity with each other (Fig 6). Certain promoters on chromosome 8 (*OsGLP8-11*, *-13* and *-14*) were distantly related to the rest of the *OsGLP* promoters present in the same chromosome. In addition to the *OsGLP* promoters of chromosomes 8 and 12, promoters belonging to chromosome 2 (*OsGLP2-1*, *-2*, *-3* and *-4*) were also grouped in a particular clade (clade IV). It was observed that on chromosomes 1, one *OsGLP* promoter (*OsGLP1-1*) was categorized under clade III while other three promoters (*OsGLP1-2*, *-3* and *-4*) were grouped in clade IV (Fig 6). Eight different *OsGLP* promoters available on chromosome 3 were scattered in clade II, III and IV. Nonetheless, two promoters (*OsGLP9-1* and *-3*) of chromosome 9, were grouped in clade IV while another promoter (*OsGLP9-2*) was grouped under clade II (Fig 6). Rice chromosomes (4, 5 and 11) containing single *OsGLP* also exhibited diversity in their *OsGLP* promoter regions (Figs 1 and 6). Tajima’s neutrality test was conducted using 1.5 kb promoter sequences from 40 *OsGLP* and it was found to be significant (*D* = 7.36 where, *D* = Tajima test statistic). Additionally, the nucleotide diversity among the tested sequences was 0.696. Most of the *OsGLP* promoters on chromosomes 8 and 12 shared the highest sequence similarity and this might be due to the duplication of the genomic region including the presence of TFbs. In corroboration to our findings, *OsGLP* promoters analyzed using 1.0 kb promoter region also depicted the relatedness of the promoters on chromosomes 8 and 12 [70]. Although, according to our study certain promoters on chromosome 8 *viz*. *OsGLP8-11*, *-13* and *-14* were distantly related, earlier study [70] depicted that *OsGLP8-11*, *-12* and *-13* promoters were phylogenetically distinct from the rest of the *OsGLP* promoters on chromosome 8. This difference with earlier study might be due to the consideration of 1.5 kb promoter region in our study. TFbs analysis revealed that the *OsGLP8-14* promoters was devoid of any NACbs and only one WRKYbs was available in the distal promoter region making it unique from rest of the *OsGLP* promoters of chromosome 8 (Fig 2). Consistent to our finding previous report also depicted that *OsGLP8-14* is preferentially expressed in panicle development stage [9], which is unique among the *OsGLP* genes. Additionally, the availability of fewest numbers of TFbs (Fig 5) in *OsGLP8-13* promoter might be the reason of the phylogenetic distinctiveness of this promoter from rest of the *OsGLP* promoters belonging to chromosome 8. Another study depicts that the promoter region of *Os RGLP1* and *Os RGLP2* gene have only 21% homology [71] and those genes are same as *OsGLP8-11* and *OsGLP8-10*, respectively (Table 1). Hence, in support to earlier literature [71] our findings can be justified regarding the phylogenetic classification of *OsGLP8-10* and *OsGLP8-11* promoters in different clade (Fig 6). According to earlier report [9] *OsGLP2-1*, *OsGLP2-2* and *OsGLP2-3* were tandemly duplicated; but as in chromosome 2, *OsGLP2-1* was in the negative strand while *OsGLP2-2* and *OsGLP2-3* were available in positive strand, they were expected to be originated from inverse tandem and tandem duplications, respectively (Fig 1 and Table 1). All of the *OsGLP* promoters in chromosome 2 were categorized into clade IV possibly due to their origin through duplication in chromosomal region along with their close proximity in same chromosomal arm (Fig 1).

In accordance with the previous report [70], in the present study also *OsGLP* promoters under chromosome 1, 3 and 9 exhibited maximum diversification. Interestingly, *OsGLP1-1* and *OsGLP1-2* were found to be located at different arms on chromosome 1, and this positional difference might be causing diversity between them. On the other hand, *OsGLP1-3* and *OsGLP1-4* were found to be located in close proximity and might be generated due to tandem duplication (Fig 1) so, these two promoters were closely related and belonging to same clade (Fig 6). In a similar manner *OsGLP3-7* and *-8* promoters on chromosome 3 were phylogenetically closer probably due to their origin through tandem duplication in chromosome 3 while *OsGLP3-4*, *-5* and *-6* were highly phylogenetically related due to their derivation through inverse tandem duplication (Figs 1 and 6). The phylogenetic differences among the rest of the *OsGLP* promoters under chromosome 3 and 9 might be due to the diversification in their TFbs as mentioned by earlier researchers also [70]. Another study on barley germin-like *GER4* gene cluster revealed that barley genome contains tandemly duplicated genes (*GER4a-h*) and different *GER4* promoters exhibited differential expression in response to biotic stress due to the available changes in their *cis*-regulatory region [14].

### *OsGLP* gene expression study in response to biotic and abiotic stresses

**Fig 7:**
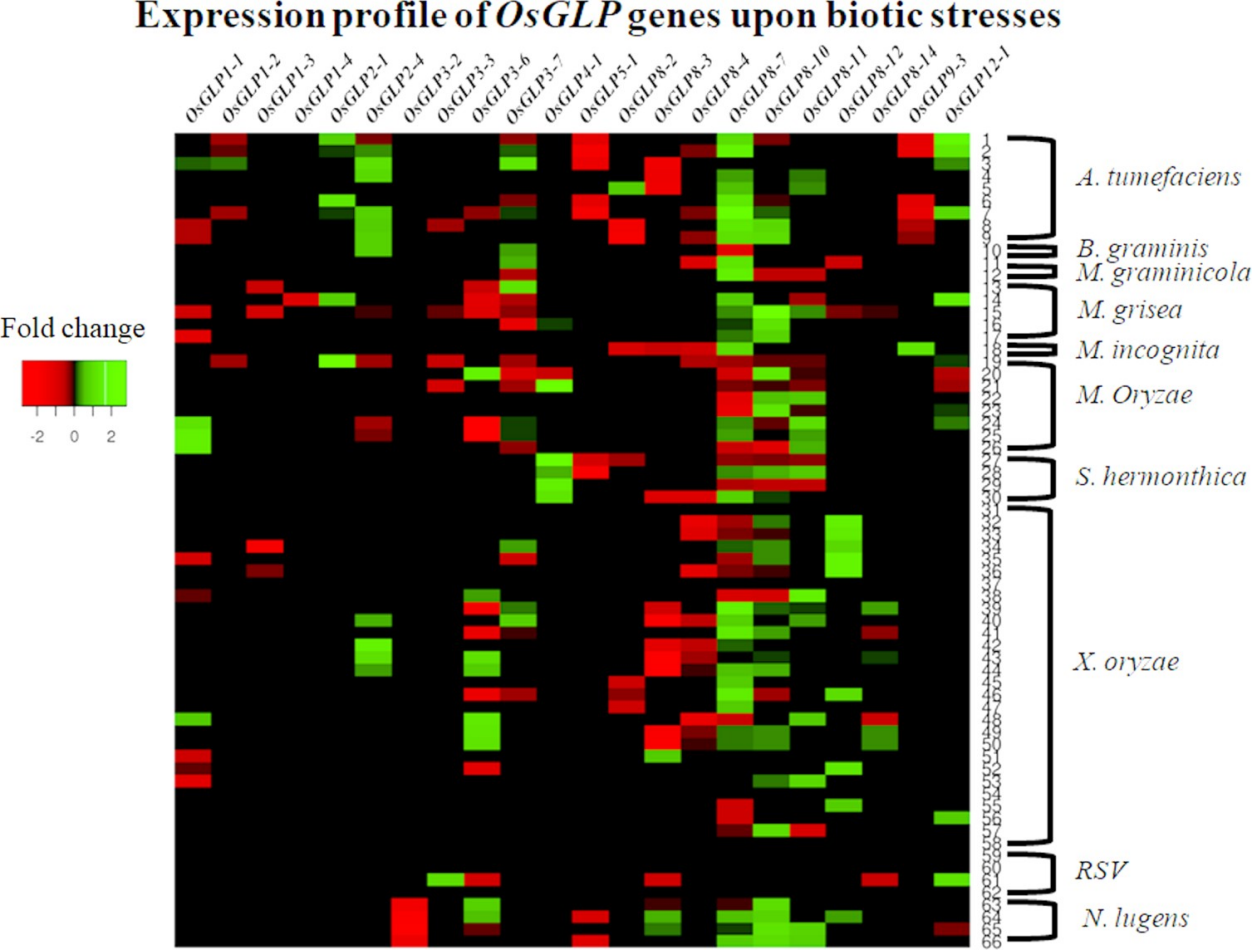
Heatmap analysis of *OsGLP* genes in response to biotic stresses. The microarray data for expression of 22 *OsGLP* genes under various biotic stress conditions mentioned in S2 File were used to generate heatmap using Heatmapper online tool (http://www1.heatmapper.ca/expression/). Color bar represents fold change in gene expression, *red* color representing lowest level of expression and *green* signifies highest level of expression.

**Fig 8:**
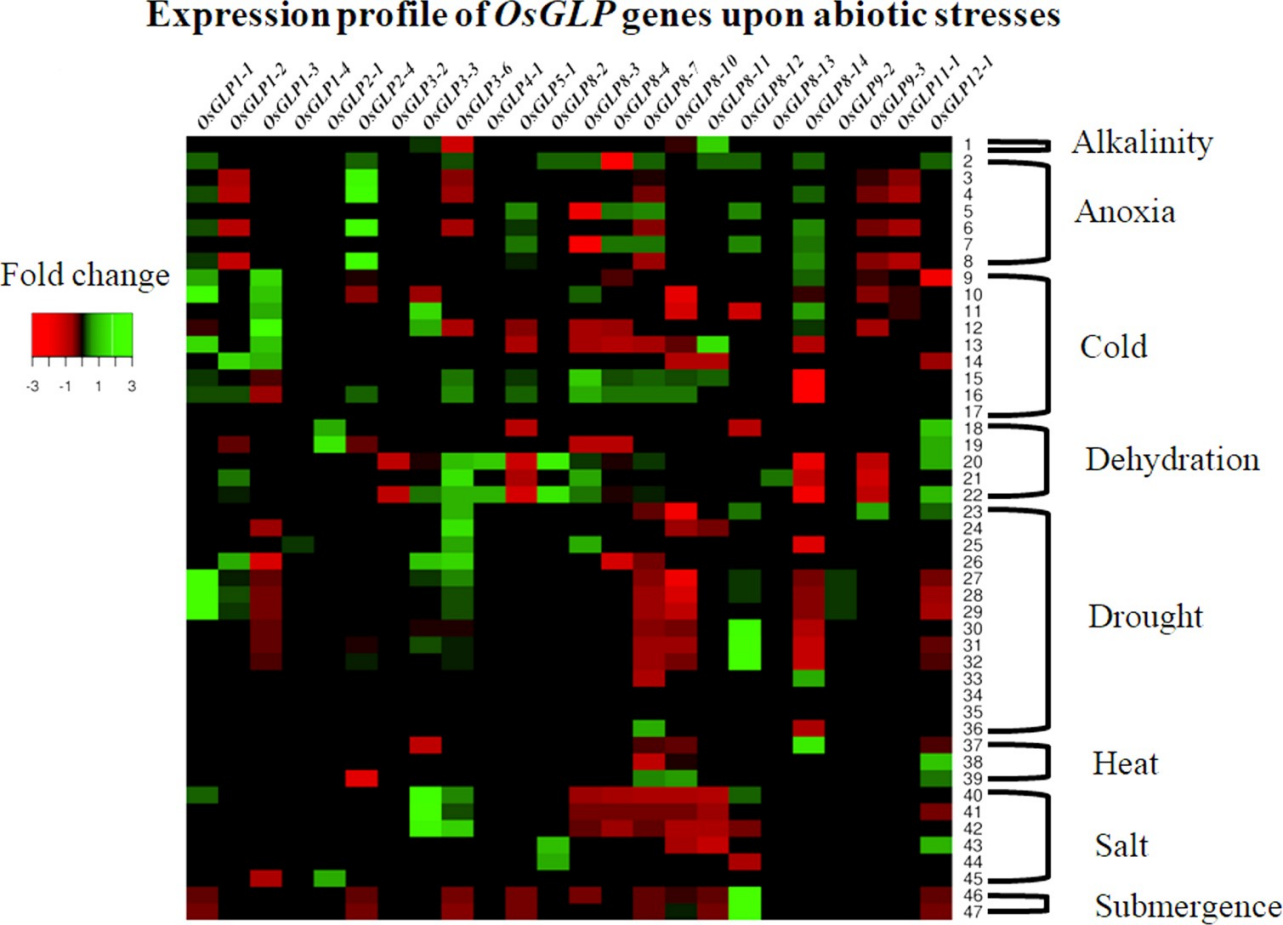
Heatmap analysis of *OsGLP* genes in response to abiotic stresses. The microarray data for expression of 24 *OsGLP* genes under various abiotic stress conditions mentioned in S3 File were used to generate heatmap using Heatmapper online tool (http://www1.heatmapper.ca/expression/). Color bar represents fold change in gene expression, *red* color representing lowest level of expression and *green* signifies highest level of expression.

Expression study by GENEVESTIGATOR using the microarray dataset from rice depicted that in response to biotic and abiotic stresses there is some correlation in *OsGLP* gene expression and *OsGLP* gene promoter classification. Gene expression of *OsGLP8-3* and *OsGLP8-4* depicted retarded gene expression under several biotic stresses while the expression study by microarray demonstrated up-regulated gene expression for *OsGLP8-7* and *OsGLP8-10* in response to brown plant hopper (*Nilaparvata lugens*), *Agrobacterium tumefaciens* and fungal infestations (Fig 7 and S2 File). Similarly the expression of *OsGLP8-7* and *OsGLP8-10* genes depicted high level of similarity upon exposure to several abiotic stresses like cold stress, salt stress and anoxia (Fig 8 and S3 File). It is to be recalled here that phylogenetic study of *OsGLP* gene promoters showed that *OsGLP8-7* and *-10* belong to cluster 1 in clade I while *OsGLP8-3* and *-4* were categorized under cluster 2 in clade I (Fig 6). Although *OsGLP8-14* and *OsGLP11-1* depicted about 3 fold expressional up-regulation upon cold stress (S3 File), due to inadequate data availability no additional significant similarity was revealed among the gene expression of *OsGLP3-7*, *3–8*, *4–1*, *8–14*, *9–2* and *11–1*. Phylogenetic study of *OsGLP* promoters depicted that all of those 6 promoters were classified in clade II. The gene expression of *OsGLP1-1* and *OsGLP3-3* depicted that upon several abiotic stresses like cold, drought and heat stresses their expressions were up-regulated (Fig 8). Additionally the gene expression data depicted that in response to *A*. *tumefaciens* and fungal infestations, the gene expression of *OsGLP1-1* and *OsGLP3-3* were down-regulated (Fig 7). Promoters of *OsGLP1-1* and *OsGLP3-3* were categorized in clade III through phylogenetic analysis (Fig 6). In response to submergence, the expression of *OsGLP3-6* as well as *OsGLP5-1* genes were found to be down-regulated (Fig 8) and the promoter regions of those two genes belong to clade IV (Fig 6). Interestingly, the expression of *OsGLP8-11* and *OsGLP2-4* also showed expressional retardation upon submergence and according to our phylogenetic study these two *OsGLP* gene promoters were also categorized in clade IV. From the present study and earlier literature, it can be enumerated that a number of *OsGLP*s located in the chromosome 8 revealed as major gene cluster conferring biotic stress tolerance in rice [6]. Similar kind of defence responses have also been observed in barley and grape by the orthologus *GLP* members [4, 72].

Although the genome wide duplication as well as neofunctionalization of the *OsGLP* genes as well as promoter region were taken place during the course of evolution, expression analyses of microarray data from public database revealed certain homology among the expression of *OsGLP* genes whose promoter regions were classified under same clade. Considering the previous finding [9, 14] and our present study it can be inferred that *OsGLP* duplication and subsequent variation in TFbs resulted neofunctionalization and these genes as well as their promoters might be involved in tolerance to biotic and abiotic stresses via broad-spectrum resistance or basal mechanism of tolerance in plants.

## Conclusion

In the present study among 40 *OsGLP* promoter regions, the presence of different kinds of TFbs was revealed in varied frequencies and some of these promoters were phylogenetically distinct in spite of their presence in the same chromosome. It can be inferred that during the course of evolution these *OsGLP* promoters were under considerable environmental pressure. In the present study we have considered six important TFs *viz*. NAC, WRKY, bHLH, bZIP, MYB and AP2/ERF, due to their significant involvement in biotic and abiotic stresses. Although certain *OsGLP* promoters (*OsGLP8-11* and *OsGLP12-3*) were found to possess larger number of TFbs and some promoters (*OsGLP1-2*, *OsGLP5-1* and *OsGLP8-13*) had fewer, further study is needed to validate these *OsGLP* promoters for subsequent utilization in plant genetic engineering as stress inducible promoter. Gene expression data revealed that certain promoters belonging to same clade had similar pattern of gene expression. It can be concluded that *OsGLP* gene duplication and subsequent variation in TFbs resulted neofunctionalization of gene and promoter regions to cope up with various biotic and abiotic stresses during the course of evolution.

## Supporting information

S1 Table Availability of different transcription factor binding sites (TFbs) in 40 *OsGLP* promoters. (DOC)

S1 File The 1.5 kb promoter sequences of 40 *OsGLP* genes from 9 different chromosomes of rice. (DOC)

S2 File Microarray data of *OsGLP* gene expression (expressed in fold change) in response to biotic stresses. (XLS)

S3 File Microarray data of *OsGLP* gene expression (expressed in fold change) in response to abiotic stresses. (XLS)
