# Supplementary material for: In-silico study of biotic and abiotic stress-related transcription factor binding sites in the promoter regions of rice germin-like protein genes
Source: PLoS One. 2019 Feb 14;14(2):e0211887. doi: 10.1371/journal.pone.0211887 (PMC6375593; doi:10.1371/journal.pone.0211887)
Supplement: S1 Table — (DOC) [file pone.0211887.s001.doc]

**S1 Table. Availability of different transcription factor binding sites (TFbs) in 40 *OsGLP* gene promoters**.

| **Gene promoter** | **Transcription factors involved in biotic and abiotic stresses** | | | | | |  |
| --- | --- | --- | --- | --- | --- | --- | --- |
|  | NAC TFbs | WRKY TFbs | bHLH TFbs | bZIP TFbs | MYB TFbs | AP2/ERF TFbs | Total TFbs |
| *OsGLP1-1* | 2 | 3 | 12 | 5 | 11 | 81 | 114 |
| *OsGLP1-2* | 3 | 2 | 2 | 4 | 2 | 36 | 49 |
| *OsGLP1-3* | 4 | 1 | 6 | 2 | 5 | 98 | 116 |
| *OsGLP1-4* | 2 | 3 | 15 | 7 | 3 | 104 | 134 |
| *OsGLP2-1* | 1 | 4 | 15 | 5 | 7 | 70 | 102 |
| *OsGLP2-2* | 1 | 4 | 15 | 5 | 7 | 71 | 103 |
| *OsGLP2-3* | 1 | 1 | 10 | 5 | 5 | 114 | 136 |
| *OsGLP2-4* | 1 | 3 | 11 | 4 | 11 | 79 | 109 |
| *OsGLP3-1* | 5 | 5 | 7 | 5 | 6 | 63 | 91 |
| *OsGLP3-2* | 0 | 3 | 1 | 3 | 5 | 80 | 92 |
| *OsGLP3-3* | 0 | 1 | 4 | 3 | 9 | 78 | 95 |
| *OsGLP3-4* | 2 | 3 | 3 | 6 | 8 | 108 | 130 |
| *OSGLP3-5* | 1 | 1 | 10 | 4 | 5 | 96 | 117 |
| *OsGLP3-6* | 0 | 1 | 3 | 5 | 14 | 86 | 109 |
| *OsGLP3-7* | 0 | 6 | 7 | 5 | 7 | 72 | 97 |
| *OsGLP3-8* | 0 | 3 | 5 | 2 | 9 | 58 | 77 |
| *OsGLP4-1* | 0 | 1 | 8 | 10 | 11 | 82 | 112 |
| *OsGLP5-1* | 0 | 2 | 0 | 4 | 4 | 38 | 48 |
| *OsGLP8-1* | 3 | 6 | 8 | 11 | 6 | 103 | 137 |
| *OsGLP8-2* | 1 | 4 | 7 | 7 | 9 | 102 | 130 |
| *OsGLP8-3* | 2 | 1 | 8 | 6 | 5 | 68 | 90 |
| *OSGLP8-4* | 0 | 9 | 16 | 3 | 5 | 95 | 128 |
| *OsGLP8-5* | 1 | 3 | 8 | 7 | 12 | 90 | 121 |
| *OsGLP8-6* | 1 | 2 | 8 | 3 | 10 | 84 | 108 |
| *OsGLP8-7* | 2 | 8 | 9 | 10 | 7 | 87 | 123 |
| *OsGLP8-8* | 0 | 5 | 3 | 6 | 9 | 55 | 78 |
| *OsGLP8-9* | 1 | 3 | 8 | 5 | 10 | 64 | 91 |
| *OsGLP8-10* | 0 | 3 | 7 | 7 | 7 | 67 | 91 |
| *OsGLP8-11* | 3 | 3 | 20 | 21 | 13 | 122 | 182 |
| *OsGLP8-12* | 3 | 1 | 4 | 10 | 5 | 54 | 77 |
| *OsGLP8-13* | 2 | 2 | 4 | 13 | 6 | 23 | 50 |
| *OsGLP8-14* | 0 | 1 | 6 | 7 | 7 | 85 | 106 |
| *OsGLP9-1* | 4 | 2 | 3 | 5 | 5 | 93 | 112 |
| *OsGLP9-2* | 1 | 3 | 8 | 4 | 10 | 82 | 108 |
| *OsGLP9-3* | 3 | 2 | 5 | 3 | 7 | 70 | 90 |
| *OsGLP11-1* | 1 | 2 | 2 | 6 | 9 | 76 | 96 |
| *OsGLP12-1* | 2 | 4 | 4 | 3 | 13 | 99 | 125 |
| *OsGLP12-2* | 1 | 4 | 4 | 2 | 10 | 107 | 128 |
| *OsGLP12-3* | 3 | 2 | 13 | 4 | 9 | 120 | 151 |
| *OsGLP12-4* | 1 | 1 | 3 | 5 | 5 | 78 | 93 |
| ***Total*** | 58 | 118 | 292 | 232 | 308 | 3238 |  |
