# Supplementary material for: In-silico study of biotic and abiotic stress-related transcription factor binding sites in the promoter regions of rice germin-like protein genes
Source: PLoS One. 2019 Feb 14;14(2):e0211887. doi: 10.1371/journal.pone.0211887 (PMC6375593; doi:10.1371/journal.pone.0211887)
Supplement: S1 File — (DOC) [file pone.0211887.s002.doc]

**S1 File. The 1.5 kb promoter sequences of 40 *OsGLP* genes from 9 different chromosomes of rice.**

>OsGLP1-1

TGGCTTGTTTTGATTGGGCATCTGCGAACTGGATTATACCCTAAAAATTTCGAAAGAAATACGAATCTTTGGGCCGGCTGAAAAAAAAGGAAGTTTAAATTTTGAATTTTTCATTGAAATCTTAGCCATTTGTCGTTGATGTAACTAACTAAGAGGATCATCAAGAGATACCCAAAAATCTATCTCAAAAGTTTTTTTGAGAATCCTAAACACAACTTGAGGTGCAAAAATATTTATATTTCCAAGAGGGAGATGGCCTAAAACTGAGTAACAAATTGAACTACCAGATCAGCCTACGATGGTCGCGATCGCCTCCTCCTCCCTGTCGATGATCGATCCTTCATGCTCTCCCCTCGGATCCGTGGATTGGAATCAGAACAAGGAGTTGTGGAGATTGAGATATATATACGGAGATGATAGGAGCCAGTTTTTTTTTTCAAAAATCCAACTAATTCTAGGACGATTTTGCTATACATTTATCGACAAATTTTTCCCCAAAAATTTGACAGATGTAATTACAGTACAATTGTAGTGTAATTACACTGTAACTTGTATGCAATTACACTGTAACTATAGTGTAACTTGTATGTAACTTTCAAAAATCTCTCCGTAACATGTTATTTCGGTAAAATGGAGGTCGTGGGAACAAATTCTTTCATATATGTGTGTGCTATGATTTTTTTCTTCCTCACCAAAACAAATCGTGCAATAGATCTAACGATTCAAAATTATGTGACACTTACATATAAGTTACACTGTAGTTACATGCAAGTTACAGTATAATTACATATAAGTTATAGTGTAATTACACTACGATTATATTATAATTACATTTGTCAAATTTTTGGTGAAAAATTTGTCGACAAATGTATAGGTAGTCCCGTAACCGTAGCAGCACGGAGACGAGGAGGTTGGAAGCAGAAAGGACGAAAGGGGAATAGATAATGCGCGCGAGCACGCGAGCACATCGGTGCACTCCTACTACTACTACCAGTCTATACCAGTGTTCTTCCACGTTGCTACACGCGGGAGTGGCGAGGCGTCCATGCCTCCATGGATCGATCGATCCGAATAATAAATACAGTAATACATATCCAGCACCCACATGCATGTGTAGCAGTTCGCCTAGATCCGCATTAAACTAGTCTATCTCCAGCAATTTATGGTTCACGGCTTTGAACTAAATAAGCCGCCTCCCGCGTGTCCACCCCTCTTGCCTCTGCTCCCTTTGTCCACTTGCCGCCTCTCCCCGTACTCCTACCAACTCTTCTCGTCGTCGGCGGCGGCTTCGTCCGGCTCACCGGCCGCTGCATCTCCCTTTGTGTCTTAGTTAGTTTAATCCCATTTACAAATCTTACGTGGTGAACTGATCAATTATTAGTTGAGCTGAGCTGAGCTTATAAGTAGCAGCTGCTCTGATCTGCTCAGCTCGCTTCATCACCTTCCACTCAGTCAATCGCAGCGAGAAGCCAAGAAGCACTCAACCACGCGCTGTTCGTC

>OsGLP1-2

TCCGCACCGCCGGTCGCCACCATCGCCTCATCCCAGATGGTGTTCACCACAATTGAATCGACGGAGCACGCGGCGTCTGCCGAGGCGGGGAGAGCTGGAGCGACGGCAACAGTGGGCGCGGACCAGAGCTGTAGCGGTGCGTCCCGTGGTTTTTGGCGGCGGCTGCGACCGAGAGCGGTGGCGGCGGACGGGCGAGGGCCGGTCGCAACGCCGCCGAGCTCGTGTGCGCCCTCCGCGCCGTCCTACTACTCCTCTACCTCCGTCGCCGCCTGCCAAAGCGCCGCCGCCTTTCGAGGCTATCGCCGGGGGTGGAGAAGGGAAAGGGGAGAGGCGGCACCGCCGAGATCTCGCCCGCCCTCTATCGCCGAGCTCGGATGCGTCCTCCACACCGTCGAGCTACTCCTCTATCTCCGCCACCGCCTGCCGAAGCGTCGCCGCCGTCCGAGAAGGGAGAGGGGAGAGGCAGCGCAGCGAAAGGTGGGGAAGGAGACGCGGACGGCGACGACGACATCGAGATGGCGAGGCAAAGCTGGAGGGGATGGTGGACGCTCCAGCGCTCACCTCCCACCCGCCACCGCCGCTCCAGCCCTCGCCCACATGCCGTCGTCGCCGCTCCAGCGCCTGCCGCCGCTATCACTCTGGCCCCGCCCGCCCACCCCCACTCCAGCCCTCACCCGCACGCTGCTGTCGCCACTCCGCCGCCGCCCGCCGCCGCCGCCGATGAGGCTCTCGCTCCCCGCGTGCGTCCGAAGAGGAAGAGAAAAGGAGGAGAGAAAAGAGAGAGAAGGAAGAGAGAAGGGAAGGAGAAAGAATGACATGTGGGCCCCCACATGCTACGTGGGATGAAAACTGGGTTAACACCGCTACGTCAGCAAAACCACCCTTCAAAATTGCTAGAGGAGTCAAGTTGTACTGGTTTTAATAGTTCGGGGAGTCGTTCTATCTTATTTTCTTGTTAAGGGGTACAAATTAGATTGGCCTACTTTAAGGGAGTTAGAGTGGACTTTTTTCCTTTAAAATGCAAACGGCGAAGAAAACTATCATGTAGATATTTCTTAATGTGATCACCTCATTCCTCTTCTCCGCGTCATTGGAAACGCGACGCAAGCTACTCCGCATCTACTTCCCGGGGACCTGCCAAATGGTGTGTCCTCCCGACCCGACGCGCCGGTCTCGACAGCGACAAACAGTACATGCCGCCACGCGCGCCTACTTGATTTCATTTGGCTATCGTCCGGCCGCACGCCATGGACGCGCGCGCTTCGCGACCCCGTCAACCGCCTGCGCTGAGCTGCAGCGACACCATCACCGATTCAGCTAGCATCCACATCGGCAGAATCGTTTCACGTCACGTCCACCCGTGCACGCATCTCGCTAGGCTAGCATTACATATAGCCAGCAGCGCATGGTCACCACCATGATCAACTCTCGAGAAAAATCGAGCAGCTAGTTAGTTCCTCACTTCTAGGGATTCGCTTTGGCTACGTACATTGGTGTACG

>OsGLP1-3

AATACAATAACATTTTCATACAAAATAAACATATTATCGAAATATGCTTAATGCTATATTTACTAAAACTAATTTGATGTTATAGGTGTTGTGAGATCTTTCTATAAACTTCATTAAGAAACATTTGAAAAAAAGGATCAAAACGGTTCTAATATGAATGAATGAATGAATAGAGGGGTACGGCATGTGCTCTGCATATACCGTTACTACGCCTCGTACGATTCTGACCGGGTAGCTAGGGTAGGGTGATTAATTATTGGTGCAAATTAACCATTGGCTATGCAGGCATGGCTAGCTGCTGTAACTAATTCTCCATCGATCATTCGATCTGCCAGATCGGTGTCCTCCTGAATTAATCTCAAATCTGGATGTAAAAAAGATAATTAATCAATTCCCCTTTCATGTTGTCGACTGCATGTGAGCGAATGAAGTTCAGCAGCTAGCTAATGCAGTTCTTCTGACTTGCAAGTCACAGGAAAGCAAATGCCTACAGTTGAGAAAAGCACAAAGCTGATATCTACATTACACAATGCTACAGTTTAATGGACGGATGATTTCTGCATTACAGCATTAGTACGTCTTAATTGCAACTGTCGGTCAACTGCTCCATTGATCGATCTCTCGTGCTGTTCCTGCATGTGCATGCATGTATGAGTACGTACGTACAGCTAGACTGGTAGTTTAATTTGATTGTATAAACTGACGATTTTAGTTAATTACTATCTAAGCGTCTATTTAGTTTGCTGTCATTTTCAATCCTATCAAAATTTAGTAATGCTGAATTTTGAAATGACAATAAACTAAACACACACTTCAACTATGTACCTTATCACAATTTGGCAAATTTAAAAGTTCTCTCTCTCAAAACTCCACTTAACTTTGGTAAAAGCATCAAACCAAACAAAATAATACTATCAAAAGTATTGTTGATTTGATAACAAAATGAACAAGCTGCTTTAAATTTATTCCTCAGGTTCTTAGTACACTGTACACCTGGATCTCTCGATCGATCCCATATACCAGTACACTATAGATTGCTCTCGCCTTAATTAATTAATTTCTAGATCTCAAGAGGGCAACAGACCCCCTGGCAAGGACAGATCGAAAAACATCGCTCGCCTAACCATTCAATTCTCACTGAAAAAGCTTAATATATTAGGAGTAGTAAGCTAGCGTGTGAGGAAGATTCATAACCATATATCATCTTAATTAGCCTTTGTGATTTAGCTTAATCACATGGCTAAGGCACAACCACCCATCCACTTAACTCTTCATTACTACGCTAGCTACACGAGGAGAGTAGCTAGCTAGTACAGGCCCCGGCAAGCATAAATAGCAGCGTCCCCTGCTTCCTTTCTTCATCGTCCTCAGCTCATCATCTGCATGCAGTTCACTGCACACCACACAGCTTAGCTTGCTCAGCTTCACTGATCTTCTTAGCTGCAGCTACTTCACTTTGCATAGTTTGATCGAACTAAATAACTCACCAAGTTAGCTGTA

>OsGLP1-4

TTTGGTGAACCATTACCATCAATCAATGTGCTTTTGTCACTAGGGAATCGTCGCATGATCGATAAGGTATTTGAACCGCCATGTATACGTAGACGATATTCTTAATTAAGTATAAGTATTCTTTCAGTTTTTCGTAATGAGAACTATATATAAATGAAAAATGGCTTGGGAAAAGTAGAATCATCTCGATCTGATACTCCAAAGTTCTTAATAATTAACTAGTGGACTACTTCTATAGATGATAGTTACCCAGGAATGAAAAAATTGAAAATAAAAAATTATGCTATTTGGAGACTTGGAAATTAAGTTCTCGATCACCTGGATATATCTATCGTTATTACTTAAATTATTTTCTGGGTTAGAATAATTATTCTTCATTTTTTTTAAGTTCTCATGGTAGAAACAACAGGAGAAATGTCTTAGGGGTCTTCCGGCTAGCTTCACAAGGTGGTGGGTTAGTTGAGCTGGATTTGAAGCCTCACCCCTTCTATTTATTTGATATTAGATTATTCTCTAATATTCACTTTTTAATGACAGAACAACAGAAGACAAATGTACATGTATCTATGTTGAATAACAGAACAAAGTGACTAAAAAAACAGGATACACAAAACCTACAACACTAATATGGCTCTCTAATATAAGTCCACAATGTGTATACTAGAATATCCTTTTTTCTTATGCCAACATACTCTACAGGAATAACTTCTGCTAGCTTGATATCTAGAAGTCCAAGTGATTTGCTTTTTGTTTAATGTTGCGAATTACCTTGTCGATTTGATTAAACAAGCTAGCTAGCGTACGTATATACGTACATTACAGTACACACTATAGCTAGCTAGTACGTAGTCAATTAGCTAGCTGCCTGCGTACGTACTTGGCGTCAACCTCAGCAGTCTTGCGTTTACGCCAGCATCTGATTATCTGAACAGGAACACGTGCACAGTATACCGTGCAAGCTACAAGGCAAAATGCATCCATCTTGTACTATTACCTGGAGTCTGGGCATTACTCTCTCTCGCTCTCCATCTCTCTAATCCCCAATTACTCTCTCTTTCATGTGCATGCGTGCTAGTTTGCATAAAACAAAACGTGCATGCCTCCATTAACAATTAACATGAGGAGTTAATTACACTACTCTCACTCTCCCCCACACACAGTCACATCGACACATGAGATAATAGACTATATTCATCAAATTTCACTCGATTTGTACATTTTTATTGGTTGCATGGCCACAATTTTATCCATATATCCATTCCACTGTCACGACTCTATTAAGCTACTCGATCCACATATCTATAAATACCACCTCCCCTAACCCTAGCTTTTCATCAAGCCATAACTTTTTAGTCAAAAAAAAAGTGATCATTGATCTAAACTACTTGCAGGTGACGCACACGTACGTACTCACACAGCTAAGAGCTACAGATAGTACTCCAGTTCACTTAGCTACATCAATCAAGGAAAATTAAGCTTAGCAACAACCAGCTAGCTTCC

>OsGLP2-1

GATGGCGCCATGAGAGCACCACAGAGCCAAGAGGGCAACGACAGCAAGCTGCTGGAGGAAGCAGGCGCCAACAGCAGCCATAGCTGGAGCCTGGAGGAGGCAGAGAGTTCTCTATAGAAAAGAATACTTATGGTATAGCAGATTGCTCTGATGATATATATATTGTGATGAACTGATGATGAAGAATGTCATGGATTGAGAGGAAAGCTGGTATTTATAGGGGGAGTTCAGGTTTCAGAATAGCCTATTGAACTCTGAAATCTCTGGTAATGATTGTGATGACAGAGAATATTGTCTGTCTGTGCATGCCATGCATGCGTCGTACAGCACCAAGAACGAGTCACTGCTAGCTAGTAGCTAGATAGGAAGAAGGCGTCATACATAAAATACATAATATTGCGTGGATGGGCAACCTGCAGTCTGCAGATACATATACCTATCTATCCTGAAATAAATCTTATTAATGTTATCATACATGCTTTGTGCTTGAAGAAGTCTAAGCCATGGATGAGTACTGTATCTGCAGACTTAGCAGCCGTGGCTATTAATGTTGTCCAAGGTGGATATATGGATCATATTAATTCTTTACTGGTTTGCTGTTTGCCGGCCGGCCTGATCATCGATTGACAATGGTCCGATCTCCATTGTAATATTCTTATCTCATCATAGCAATTAAAATTAACACCATATTTGTTCCGGAGAAAAATGCACGTAAGGAAATAATAATGCTTGCTTTTCATTGCTTGCTTCCTTCCAAGAAGACGGGATTAAAACTCCTTCGACCAACCGAAATGACAGAGGATTGACTACACGCCTATACTATCTACAAAATTATGCCTAATTAAACGTTGGATATAAGAGGACGGACTCCCATATCAAAATTTATTAAGACTCTGCATGCCTTGAAACCGAGTACATCGACTAATCCACACCTTGTTGTGGTGCTAACTGCTGCATGCCTTTCTCAATGATAATGATACCTAATTAAAACGTAGTTTTCCCAAACAGAATTTGTTAACCGAACGACGAACGCGTAATGCATAGCCATTATTAAATTGTTGCTTGACGTAATTTGTTTAAGCAGGTGCCGTTACCATCGAGTCCACCTGGAGAGAGCACAATTTACACATGATGAAGAATCAGTCAGAAGCTATATTAGCTTCTTACTGAATTTGCTGTAGCCGCTGCTGCAGGCAGGCCACAACACGTTTATTCAGACTTGTTCCAAATTACCAGAGAATTGGCGTAGACCATTTCTTCAAACCTCAATACATATTAAACATCCAATTAACTTTCTCAACACAAAGCAAGCACAAGCCAGCTTGATCCTCCTACTCTACTCCATCTCTATATATACTGGGTATCTCTCACCCCGTTTGAAGCACACAGCAAAGCATCATCATCAGTTCATCACATCACAAGAAACTTTGCGTTGCATCCTTTTGTTTCCTGCTAAAATTGACACGCACTTGATTTAGTGATTAGTGTCCTAAACTCCTA

>OsGLP2-2

GAGTTGGCTGGGATCGGAGGCGAAAGCATTCGAAATCGATACAGCCAGGAGGGCAAGGAGGAAGAACCATGTTGAGGCCATTAGGAGTTTAGGACACTAATCACTAAATCAAGTGCGTGTCAATTTTAGCAGGAAACAAAAGGATGCAACGCAAAGTTTCTTGTGATGTGATGAACTGATGATGATGCTTTGCTGTGTGCTTCAAACGGGGTGAGAGATACCCAGTATATATAGAGATGGAGTAGAGTAGGAGGATCAAGCTGGCTTGTGCTTGCTTTGTGTTGAGAAAGTTAATTGGATGTTTAATATGTATTGAGGTTTGAAGAAATGGTCTACGCCAATTCTCTGGTAATTTGGAACAAGTCTGAATAAACGTGTTGTGGCCTGCCTGCAGCAGCGGCTACAGCAAATTCAGTAAGAAGCTAATATAGCTTCTGACTGATTCTTCATCATGTGTAAATTGTGCTCTCTCCAGGTGGACTCGATGGTAACGGCACCTGCTTAAACAAATTACGTCAAGCAACAATTTAATAATGGCTATGCATTACGCGTTCGTCGTTCGGTTAACAAATTCTGTTTGGGAAAACTACGTTTTAATTAGGTATCATTATCATTGAGAAAGGCATGCAGCAGTTAGCACCACAACAAGGTGTGGATTAGTCGATGTACTCGGTTTCAAGGCATGCAGAGTCTTAATAAATTTTGATATGGGAGTCCGTCCTCTTATATCCAACGTTTAATTAGGCATAATTTTGTAGATAGTATAGGCGTGTAGTCAATCCTCTGTCATTTCGGTTGGTCGAAGGAGTTTTAATCCCGTCTTCTTGGAAGGAAGCAAGCAATGAAAAGCAAGCATTATTATTTCCTTACGTGCATTTTTCTCCGGAACAAATATGGTGTTAATTTTAATTGCTATGATGAGATAAGAATATTACAATGGAGATCGGACCATTGTCAATCGATGATCAGGCCGGCCGGCAAACAGCAAACCAGTAAAGAATTAATATGATCCATATATCCACCTTGGACAACATTAATAGCCACGGCTGCTAAGTCTGCAGATACAGTACTCATCCATGGCTTAGACTTCTTCAAGCACAAAGCATGTATGATAACATTAATAAGATTTATTTCAGGATAGATAGGTATATGTATCTGCAGACTGCAGGTTGCCCATCCACGCAATATTATGTATTTTATGTATGACGCCTTCTTCCTATCTAGCTACTAGCTAGCAGTGACTCGTTCTTGGTGCTGTACGACGCATGCATGGCATGCACAGACAGACAATATTCTCTGTCATCACAATCATTACCAGAGATTTCAGAGTTCAATAGGCTATTCTGAAACCTGAACTCCCCCTATAAATACCAGCTTTCCTCTCAATCCATGACATTCTTCATCATCAGTTCATCACAATATATATATCATCAGAGCAATCTGCTATACCATAAGTATTCTTTTCTATAGAGAACTCTCTGCCTCCTCCAGGCTCCAGCT

>OsGLP2-3

GATCAACATTCGTGTGTAATACAAATGAGCCCCTTATATCTGGCAGTCAATTTTGAATTTTAACAAACAGTTTCTACTATCAGCTATAATAAAATACTATTACCATCATTTAATTTCCCAAATTCCTCCATATATGTACATCTAAATATTAAAAACAAATCCAAGACAGCACTGGGGTAATTATATATTAAGGTATAACAATTTGGTACGTAGATGTAATAATTTAGTATTTTTAAACAGATAGAGTACTAGCTAGTAGAATACACATCATATATTCAAAGTTTATAATTCTCTCCTAACGGTACAATAGAGATAGTGAAGTGGTCTCTAAATTATACTTCCTCCGTTTCGTATTATAAGCATTTCTAGCATTACCCATGTTTATATAAATGTTAATAAATTTAGAAATGAGCAATGCTAGAAGATCTTATAATATAAAACGGAGGGAGTACATACACAGAGAGATCGGGAGAAAGGGTCTTTAATTCGCTAGCGCTAATGATATATATATCTCCATCGGAATATGTCGACATACGTAATTAATGTACACTCAGAACATGTCGAACCTGCAGCAGCTGCGGCACACTGTTAGCCAACAAACATTCAAACAGCAAGTAAAGCGAACACGGAAAAGCTCTCGTATAAGCGGATGAGCCTGACAAATCGATCATGAATCTGGTTTAACTTTTAACGCTGTCACGCATCACCATATGAATGCATGCCGTGATCCAACTATCCTTCATTGAACATAAACTAATTTAGCGTCGTATTTCCTAATTAATATATATAATTTGTCGTCATTATACACCGTTGTTAGCCTCCAACTGGAGGTTATACGTATAGTATACACTTAGCTTTCTGTCGTGAAATGCATGCGACCTGTTCAACAATTATTAGTTTCCTCGTAATTATTCTATTCTAGCCGTCTTCGTGTCTATTATATCCACATGATGCATGCACGCCGGCTGCACAGATAATTTGGGAGTACCAAAGAAACTACTGCAATTTTTTTTGAGTAAAAGAATTTTTAGATGTAATTATCGTATAATTGAAATGTAATTATATTGTAACTACATTTAACTGTATTATATCTGTATTATAATTACGATATAACTTATATAAAACTTGCATTCAAACTATAGTTTGGTTAGCTGGAACCGAGATCTTACATGCGATATGTGTGAAAATTACTTTCTAGTAATTTTTTCCTTCATACACAAATCTTGAGGCAATCCGATGATCACAAATATAAAAATTTTAGGGGCAAAAAAATTTGCATAAGTTTTCTAGCAAATTAATAACAAGATCGATCGATTTGGCTTCCTAGGCCCTAGCTAGCTTAATCAGTTTCTATAGCTAATCCTCCCTATAAATAGGCTTCCTCGCCATTCTCACTGATCACACACATTCACAGAATTTAGCAAGTTAAGCTCGTATAGCTGCTTGCTAATTAATTTATGTTAATTAGTAGCTAACCTAGCTGACCGATCGAACGAAGCA

>OsGLP2-4

TGATGCAGGAGTGCGATTAAGTTGCGTCAGCGGCAAGGGATTATTATCCCTTTCTATATCCGGCGGCGGCGCGCTCCCGGCCGGTTGGAGGCGGCCATGGACAGCCTGCTACGCCACGAAAGCATCATTATATCTGCCCAGATGATTCATACACATATATGATCTCAAAAGGCGATCGATGTGCGCACGTACATACAGGTCACAACTGGTCATGCCTACGATAGATATGCCGTGCAACATGCGTTGCTCGGAGCACTTCAATTAAGATAGAGGGAACAAATGTTATAAGAAAGATTTTCATCGGTAGTCGACTGTTGAGAAAAGATGTGCATTCTATGTATAAACACAAGCTACTGAACTGAAAGCCGAGTGCCAAACCGGTTATTGGTATTATTCATGATCATTCCCAGATTATTCTGTTTTAACCAGTCGTGGGAACTATTGGCCAACTATGCTGGATCCTATGACTCCTCCCTGGTCCTGAGTTTCTTGGAAGCCTTATCTACAGGTTCAATGCACGCACGCACGATTGACGACGACCTGAACGACTCTTCCGGTCAATAATGCCAGCCCATGTGGTCCTCCGCAGTCCGCACTGTGCAAGTGTGCAGCGTCCACTTGAAAATCCACAGAAGCCACCATTCTTCAGAGCCTCCCTCCCTGCTTCTTCAATACACTTCCAGAACTAGGGGCATGTTCGATTTAGCCTGGTGTAGTTGGAGAAAATTTTTAATTTTAGTTATCATATCGGATATACAGATATATATTTGGAGTATTAAATGTAGTCTAATAACAAAACAAATTACAGATTCCGTCAGAAAACTACAAGACGAATTTATTAAGTCTAATTAATCCATCATTAGCAAATGTTTACTGTAGCACTATATTGTCAAATTATGGCATAATTAGGCTTAAACGATTCGTCTCACAATTTACACGTAATCTGTGTAATTGGTTTTTTTTCCTACATTTAATACTCCATACATGTATTCAAACATTCGATATGACGGCACGAAAATTTTTGTTTTGGGAACTAAATAGGGTCTTAGAGGAAAAGTAAAGAATTTGGATTTCTCAGGATTAATTTCTATATGAGTTATTCGATTTGTTAAAATAAAGTATGAGAAAGCCAAAACAATATTTTCTTTCCTACAAGTTGCATAGAGAAAAAAAAATCCACTCAAACCTCTAATTTTTTTTCTATAGATTGATATGTACATATATTTCTATCCCTTCACTTTTCCTATTCTTTTCCGCATGAAGAAAAAAAAGAGAACTTTTCTAACCATCTCTGCACTTCCGGGAAAGCGGCCGATCGACGCTTTACAACTGCTCGTGCATGACAAAATACAAGAATAACCTACGGATCAATACCTGCATGATTACTGGCAATGATCAACTCACCTTACAAGACATCAGTGCTCGACTACATATGTACTACTACGTGCAAAGCAAGCAGCTGTCCACCGACGAACGAGTGACAGAACGAGCGTAGAATCG

>OsGLP3-1

TATTTCCCTTCCAGAATTATATTGGAAGGGATGTTGGCATCAAATATCATGTGAAAGTCTAGATTCTAGAAATATGATATATTTTTTTCTCAGAATTAAAATGTGATTAGGATGCTTAACTATATTTCCCTTCCAGAATTATATTGGAAGGATGAACCATATGAACACACCAGAGTTTGTCAAGGAGTTTGAGAAGGAGCACCAAGGTCAGCTTGCATTTTTCTCTTTATATTATGCGTGTCTAGTTACTGACTTCTGACTAAAATTTTAGTTAAATGGCTGGAGATCCATGGACGCATACGGGATATGATACGATGTGTCTTTGAGTCAGCCACAGCTGTTCATCCTGAGATGCAGAACCCGTTCTCTAGGGCTATCTATGGGGTTGATGTCATGCTTGATAATAAATTCAATCCGAAGATTTTGGAGGTATGCCTTACCTACTTTCCTATCCTGCAACCGCTGAAATTTCGTTGCCTCCCCCTATTGTTGACATGACATGCTGAAACCTGCATATCTACACAACAATTGTAGTGCCGCGGCACATGGTTAAAGTTATATAGTACTGACGTGTGAAAGCCATTGCCCATATGCTTCACTGAACTAATGAAAGCCCTTGCGCATTCTCATTTTATGCCCAGCTCAGAATTAATTTCAGAAGATATGCCAGTATGTTGATTTAGATATTCTTGTTGTAGCCTCACATACATAGTATGTCAGGCAAGATGTGGTACAAAAGTCAGGAAGTAACACTCAGTTAATCTCTGTTTGCCACCCAAGCATAAGCTTCCAATTGAAATTTGACTATTACATTTGGAATTTTATAGATGTTCAGCTATTGTATTATATAAGCATTAAAACCTTCTTCCTAGTAATTCCTCTCACTGTTCCGAAAATTTATAAAATGAAATGCAGGTGACATATTGTCCGGACTGCACGAGGGCATGCAAATATGACACTCAAGCTCTCGTGGGAAGCCAGGGTGTCATCAGGGGCACTGAATTTTTCAATACAGTGTTTGGCTGCCTCTTTCTGGACGAGCTTAAAGATGTATCGCCGCTGTAATCACCACAACAAGGTTGCTGTAACACCGAAGTTCAACACAATACACGCACATGTGAGTTGTGATCCTGTCGTTTTATATGCCATACATATCAAATTTGCTCAGTACAATTACAAAGGGGGTGTGATGCTGCCTCCTGCCTGTACCATGCTTCGGATCAGATTGAAATGAGCTTGACGAATCCTCCTCCTGCTGTTCAAGGTTTTGGTTGGTCAAAAGCCAGAAAGCACGTACCTCATGCTCCAAGGCAAGCTAGCCCTTACCTTTCCGCTACTCATACCCACAGTAACTTGAAAGCATCATCACATGGCAACAACATTCTAGACATATATACACAGGTAGTAGCAGCTACTGATATCTCGACCTAATCATCTAGAGAGTAGAGGCATATACTCCTTTCCCGCCTAAACTGGAGCCAACCTGATCGAGACGCAGCC

>OsGLP3-2

TCCTCCTTGGAGATGTTACCTTGTGGACTCTCTCCCTTTCGGGTGAAAAACCAGTCCAGTTTTTGGGCGGACGTCGGCAGCGACCTCTGTCGTTGTTCACCTCCTTGGAGGCTTCGCCTTGAAGGTTTTACTCTTCCCTCAATCATTTCTGTGGGACTAGTCATTTTGTCACAGGCTTTTTATGTCTTGTGTATAGTTTTGCCGGTTTCCTTTAGCAAACTGTGCAGTTGTATGGTTTTCAGACCCAGTTTTCCTTATAAAAGGATCAACTCTCTTCTTCTAATATATCCGATAAAACTCTTACCGTCAACTGTTTCAAAAGATTAGGAAAATATCGATGCAATTTAACCAATCGAAGAACAAGCCTATCTGGTACGTAAAAAAAGAGACCACCTTGTCGTCGACACCAACGGGACCTCGTACGTCGTGGGCCAGCTCGCCGTCCGTTGTGGCAGAGAGAGATGCTCGAGTCCCTACTGTTCACGGGGGTGACGAGGTTGCCGTCCAGACGACGGCAGTACTGTCCTTGACAGCGGTGAACCATACGGAGAAGGAGAAGACATTGTTGCCGTCGTCGCCCACCTGAGTGAAGCCGCAGCTGTAGGAGAAAGTGCCGTACGAGAGTACCGGAGCGTTGCCCTTAAATTTAGGGGTTGTTTAGATGGTGCTAAAACCTTTTCAGTCTCCGTTACATTGTATGTTTGGACGCTAATTTGAAGTATTAATCGTAGACTAATAAAAAAAACTAATTTCATATATAATAACTAATCTGCGTGACAAATTTTTTAAGTCTAATTAATCCATAATTATTAAAAGTTTACTGTAGCATCATATTGTCAATCATAGCATAATTAGACTCAAAAGATTCGTCTCGTGAGTTAGTTAGGAAATGAGTTTTATAATTAGTCTATATTTAATACTCTAAATTAGTGTCTAAACATCCTATGTAACAGGCAAACAACCCCTTATTCCTCATGTTCTTATAGTATACTGTACACCTGGATCTCTCAATCGATCCCCTTTACCAGTACACTAAACATTGATCTCGCCTTAATTAATTAATTTCTTGATCTCAAGAGGACAACAGACCCCCTGGCAAGGACAGATCGAAAAACATCGCTCGCCGAACCATTCAATTCTCACTGAAAAAGCTTAATTAGGAGTAGTAGCTAGCGTGTGAGGAAGATTCATAACCATATATCATCTTAATTATTAGCCTTTTCTGATTTAGCTTATTCACTTAACTCTTCATTACTAAGCTAGCTACACGAGGCGTAGCTAGCTAGTACAGGCCCCAGCGAGCATAAATAGCAGCGTCCCCTGCTTCCTTTCCTCGTCGTCCTCAGCTCTCATCTGCATGCAGTTCACTTCACACACAACACGGCTTAGCTTGCTCAGCTTCACTGATCTTCTTAGCTATAGCTACTTCACTGTGCGTGCTAGCTAGCTAGCTGCTTTGCATAGGTTGATCGAACTAATTAACTCACCAAATTAGCTATA

>OsGLP3-3

CACCTAAGGTAGATAGTCTCTCTAATGTTGTGTTTGAGGAGAAGGGATTGAGGAGATTGGGAAGATACGCAAAACGAGGTGAGCCATTAACTCATGATTAATTGAGTATTAACTATTTTAAATTTCAAAAATGAATTAATATGGTTTTTTAAAGCAACTTTCATATAGACAATTTTTGCAAAAAACACACCGTTTAGTAGTTTGGGAAGCGTGCGCGCGGAAAACGATGTGCATTCTCACTCCCTATCACCCAAACGAACGCAGCCTAACTCTCTATACAAGGATTTCGCAGTGGATTTCCCACCGTTCCAAATGAGGCTGTGTTGATCTCATTTAACACTACCTCTTCTAGCAAACTTTTCAGCCTTTTCATAGCTCTCTTTTAAACCATAGGTTCCTTCTGAAATTCAAGTTCCATTCCATTCCATCACACTTCTCTTACATTGTTTTCTCTATTATTTATCTTTGCACGCAAGTAGAAATTGGGCGTCGGTTTTTGCAGAAAAAATCAGGCACTCCTCCCTACTGCGTTGTTGTTGCTTGTGCCTCACGGTTAATCGCAAATGAAATGTAATTTACCTTGCTCTCTAGACTTGGCTGGCATTTCTCGAAGGTTGGCTAACAAATTCAAATTACTGGTTGTTTTGATGTTTCCTTTGGCGGTCTGTGCCTCTTAGAAAAAAATCTTGATCCCTGTACGTAGGGATCGATATGCATTGCCCATGTAGCATGCTCGCCTATATATAACGCAAAATTTCTTCTAGGACACTCTAATTTTTTATCAGCTTTACCATAGGACATTTGAGTTTTTGTGTCCCATCCTTTTGTCTTTATTGTCAAAAGATATTCTGACCAATATGTTAACATATCTTTGTCCATTGTAGATATGAAATTTTGGAAAAGATGATTTTATCCTTGGCCCACCTATCATGATTTAAATTTAACCAAATTAAGTTGAATTTAAACATATTTTGCAAAATTCAAACCATGTCCCAAATGACATAAATGACAATTGGAAAAGAAATTAAAAAATAATAAAATAAAATTTAAACAAATATTGGCTGAATTTAAACAATTTTGCATAAAAAGTTTTGGATTTCCACAATTCGGGAAATGTTTTGAATCGTCCACAAAACATTGTTTATATTCAGCCAAAATTTGTTTAAATTTAAATCTTTCATGGTAGTCGAAGGGTAAAACGTCTTTTGCAAACGTTCTCTTTGGATTAGAGAGGGTATATATTAAAATAATTGTCAGAGCATCTTTTTACAATCAGAAAATTAGAGCGTCCAGAAGCAAACGCTAGAAATCTAGAGTATCGTAAAGCAATTGTTGCCCCTATATAAAAGGCTCCAAAGCTAGCTCGATCATCGGCACCCAATTAGCAAAGTAGCTTCTATACTACTGCCTCTGCCAGGTGCCATTTCATTACTCAAAGAAATTGAGCCGAGACCTGAGGAACATATACAAGAATTGGCTAGCTTTGCTCGTTGATTAGCA

>OsGLP3-4

CACCCATCCCTTGGATTCCCTTCCCCCAAACTGACATCTCATGCTTGCGTCGACAATAGTATGGAAGCACCGTGGGGAGTCTGGCTCTAGCATTGTCTCACCACCGGTGTGGATTTGATCAGCGCGCTGCCTGAGGAGCTGCGCCTCCCCATCCTCAACCATGTCCGTTGCACCTGAGCCGCTGCCCGCACAGGTCTGTTGTCGCGCAAAATACCAATTGTACTACCTCCGTACGAAAATTTAACAGCATTTAGCTATAAATCTCATAACTAACACTTGTTATATTTAAAATTGAGGGAATAATTTCTATTTAGCTCCTAGTAAATCAAACTAGAAAACTTACCGCATATATTTATATCTGTGCGTCTAAGTAAGCTGAATCCAGCTAGCTAGATAGAAAGATTGTCTGGTGTGAGTAGTTCTAAAAAATGAGAACCAAAATTCTTATTGACTACTACATACTCCCTCCATTCCAAATTGATCTACATATAGTTTTTTTAAGGTTATTCCTAAATGATCTACATATTTGTGTTTATTTATTAAGTCTATTCGTTATTTGTGCATTGGAGTAAATGGATATTGATGCATGTATCCATGTACACAAGTATTTATAACCCACATGCAATATCTTGATTTGCTATTGGCTAGAAAATAGTGGGGATGGTGTATGCATTGAGTTTGTTGCTAGAGTAAATATAGTATGAGAGAGTTATTAGTTTTTCTTGGTCTTGGTGTACCTATGAAATATGTAGATCAATTTGAAATGGATGGAGTAGTATATAAATCTACAAGCATGTTTCATCCTTTGAACAGTAGGCAAAACCCAGCCAGCAGCCACCAATAATCGCCTCGGCGCAGCAGGTGTTTCTTGCCTGGTTATCTCTTTCAAATTCTAGAGGGTATAGCTCTTGGGCTCTTGGCTGGTGTGCTTGGCAGAGAGCAAGAAACGTCCACCCGGCGGCTTGTCGAGCCCTCCTGGACCAAACACTGAATAGATAATATTGAAAATTAGTAGAGAACTAATTAGGGATAAAGTCATTATTCCTCATTAGAGAAGAAAATAATTGAATAAATCATCTGTATTTCTGAGAGAGGCATGCATGGAGTATGTAGGAGTATATATGTTGCTAGCTATATAGGTCCTGGCTGATACAGTACAGGCTAGTCTCTTACTATCTCTATTCCTTAGCAGCCTCTTTATGGCAGTTTGACTGTTGACCCGTAGTTTTGTCACATGATGGATTTCGATGCCACACTAATCTTCCCATAACTGAATTGTTAATTTTGTCACGCGAGCAACGCAAAACGGCAAAATGAATGATGCATAGCTCGATGTGCATGGTTGCTTCCCTATATAAAGGGCACCATAGCACCAAAGCTAGGCTAACTCATCAACACCCAAGCAATTTAAAGTAGCAAATTAACAAGCTAGCTAGCCAGCTCGTCTCCTCACTGCTACTGTCAGCCTGATCGAAGAAGTAATTCGCTAGTTGATTGGCA

>OsGLP3-5

CACAACATATGCATCTATCATTTCTATAATATATAACATTCATTTATCCACATATCTCACAGCTAGCAAAGCGTGAGGTATTAACTAGTATCTTATGTAATTGTGATCAAAAGCTCGACAAGCATTGAATAATATCTATTCTATGAACTTTATTGGTTTAATAATTTCAAGTTTATAAAGTTTAAATATTCAATTAAAATTGAAAGTCTACTGATCTCCCCGCCATGTAACTACCGAAGAGTTCAAAACTTTTTTTTTTCTGTTAGTTTTTATAGCTTGCATATCCTTACAAGTCGTTTACTTGTATAGAGCAATCTCGTAAAACAACATAGGGTCTAATCTAGTAACCAAGAATTTGATAAGGAGGACTCACATGCAGGGGTGAGGCAAGCTAGGGAAGCTTGGGCTACCTCCATAAAAAATATAACAACTGTACATCTAATAGTAAACACTTGCTATATTTTGGAATAGAGGAATAGTTCTTGTTTAGCTCCTAGTAAATCAAACTAGAAAAGTTACCGCAAAAATTTGTATCTGTGCGTCTAAGTACAGTGAACCCAGCTAGCTGGATAGAAATATTGTCTTTTGTGAAATAGTTCTAAAAAATGGGAACCAAAATTCTTATTTGCTACCAAATAGCATATGAACCTACATGCATGTTTCATCCTTTGAACAGTAAGCAAAACCCAACTAGCAGCCACCAATAATAGCCTCTGCCCAGCAGGTGTTTCTTTGCCGGGTTATCTCTTTCAAACTATCTAGAGGGTATCAATTGGGTGGTGTGCTTGGCGCAGCTTATTTTTTCAATGTTTTGCTATTATCCCTTTAACACTTTCATTTCTATCAAATCCTATATTTTTTATTCCTTCATTTTCTCAATTCCGTGTTTCAAAGGAGCTCGATATAGCATTAAATGTTTACGTTTATGCTCACAAGTTGTACTTGTTATAATAAATTGGAAGCTTCCACCGGAGCTAATGTGCAACTCGTAGGTATAAATAGATATATTTAATTATTTATGGTGAGAAACCTAACAGTATATACGAATATCGTCTCTTTGAGCAAACAGTGCTAAAGATAACATTAAAAATCAAAATCATCGGGGGAGAAGTCATTGCACGTTAGAGATGAAAATAATTGAATAAGCCATCTGTATATCTGACCGGCATGGAGAGCATGTAGGATTTGTTGCTTATAGGTCCTAGCTAATTTCACTACAGATCAGTCTGATCTGTTCCTTAGCAGCCTCTACGGGGTCTTGACTGCTGACTAATCTTCCTATAAGTGAATTGCAAATTTTCTCACGTGTGCCACGTACGCATGAAACATGATCAGTTATGCACGGAAGGCACGATCGATGCATGGGTGCTTCCCTATATAAAGGGCTCCAAAGCTTGCTACATCATCAGCAAAGCAAAGTAGCAAACAAAGCCAGCCAGCTCGTCACTGCTACTGTCTGCCTGATTGAAGAAGTAATTAGTTACTAGTAGTTGATTAGCA

>OsGLP3-6

AGGTGCATCTAGTCGATGCACCTGACTATATTTTTCAGCCCTCCACAGTCTTCAGATTTGGTAAAAGTACTCGGGAGACCATGGCGAAGATGATTGAAGCACTCGGCTTTGGAGTAATCTAGTTCGAGAGAAGGTTATCTTTCCGACTGTGAAGGTCTCAGGGGCTACTGTGGAGATTATGGGTACCCCATACCCACACGGCATGGTTGTCCGACTAGTTATAGGGGATAACTTATATCTATGTAATATGTAACAGATCATGACTTGGGTGTTACGTTTCCTTGTATATTACGGAACGGCCTAGAGTCCTGATTTAGGAAACCGATACCGTATTGGTTAAGGTTTCTATCTTGTAATCCTGCCCCCCATCCTATATAAGGTGGGCAGGAGGCCCTCTAGGGGGCATATATGAGACAACCTGATCGTCAGATCAATACATACTCGGCGGATTCAAATCCCCAAACAGGAGTAGGGTATTACCTCTCATTGAGAGGGCCTGAACCTGTCTAAATCCTTTGTCTCCACATCCATCCACTTTTAGGTCTCGTGCGCTACCCCTTTTTATTATTGCCGAAATCATGTTTCGACACCCAGTCGATCATCGGCTTATATCCAGTTTAAAATTAGACAAATGTAATTTGTTCTAGTGAGAAATTTGACCGAGATTCGAATTAAAGATCGATAACATTGATGAGAATTGAAATCATTTCGTTGCTCTAATCTATAGATGAAAAATAGCAGCAATGGAATAAATCGTTAGGTGGGGCGTTACAAATTTGGATGGCAAAATAAACGAGCCCTGAACGCCACCAGCAGGGTGCATATCAGCATACGCCCATCCCGTCGATCTGCGTCCAGCAAGCAGACACACGTAACCAACTGCGACTTACTTTCTTTCGTGATAGAAAGAAAGAAAGAGAGATAAAGAAAAAAAGAAGAAGAAAAAAATGAGAGAGACACAACTGCAACGTACTCTCTCTTTCTTTCGTGATAAAAAAGAAAAAAAGAGAGAAAAAGAAAGAAAACAAATAAGAGAGAGAAAGAACATCAAATCTCTCTTTCTGATGGAGATTTAGTCCATGCAGATAGGTTCTAGCTAATTCACAGTCACAGGTTCTCTTCCTTAGCAACCTCTTTGTGGCCTCGACTGCTGACCTGATTTTTCTCACAAGATTGTTTCGACGCATGTAACAATAATCCGGTAATCCCACAAAACTGCACTGTTAATTTTATCACGTGTCCCAACATAAACTATGCATTTCCTCGCAAAAAAAAAGAAAAAAAACATAACCTATGCATGACTCATGCATCTGTGCTCCCCTATATAAAGGGCTCAAATGCTAGTTCATCCTCATCCTCACCAAGCAAAGTATAGCAGCAAACAAAGCCAGCTTGTCACTGCGCTTCTTTCCTAGTTTCCTTCTCAATTCTCATTCTCAAAAGAGAATATATTGAGCTGATCGAGCTAGATATATAGAAGCACTAGCTAGTTGATCAACA

>OsGLP3-7

ATTCTGAACTAAAATCCTGGCCCAAACTCATGGATGGAATGGGATTCTGCATATTCCAAAGGCGGCCTTAGGAAAATATTTGAATTGAATCGGTTGTTCAGAGATGTGTCTTTGTAAATTTGGTGAGAAATGTATGACCCAGTGTAGTTTGTCTTCTTGGAAACATACTGTACCATCTTGATACGCGTAGACTGACTTTGTGCTGATTGTTCATCTGAAAAGAGTATTGTCTTCCAAAGGCTGATGATGAACTCTGAAAAACTGAAGATTCATCTGTACCATAGTAGCCTAGCAACAAGCCAACATGCACTGAACACGCGCTTGCTACCTGCCAACCAATTTGACTTGTGCTTACCTTTCTTCCTACAGTGTGGTCGCTTTCGCCTTTATTTTTTTTAGTGGTGGGTTCGCCCAAGTGCACGGAATTGAGGACTTCGCTTACCATTGGAGAATTTGGTCTTCATTGTTTTTAAAACTTAATTTTGAATTTGATTATTTTTTATTTTTCATCATAGTTTATTTTGAGTTTGCTAATGATATGCATATAAAAGTTTTAGTCACAAATGATCTTTTATTTAGTACTCTCTCCGTTTTATATAAAAAATATCAGCATCTAAAATATCAAATTAGTTTTATTAAATATATTTTAATAATATATTTGTTTTGTGTTTAATATACTACTACATTTTTCTATAGATTTAAAAAGAAGTTTGACTAGTAAAAAAACAAAATGATTTGTATATGAAACGGAGGGAGTAATAAGTGATTCGAATTAACATAAGAATAAGTAAAACAATATGCTCATCTCGCACTCCGTGACCAGGTCTATTCTACCAGACCACATCCTGTCCACGTCACTCGTTAGTCCGTCAACGATGAGGACGATTTACCAATCTCCACTTATCATGCAGCCGTCTAATCGCGATCCGACGGCCAGGATCATCCGACGCGCTGCCCCTTGACAAATCGGACATGGTTGAAAAAGCTTCTCTCACAATCTAGTTTTGATCACATTTTACCAACCACAACTTAAATACTTAACAACTCTCCAATAAAGTAATGATCAAAGCTTGATGATGATTATGCATGGAAATCGCCCGAGGGCCTGTCAATTTCTCACTCTCGCTATAGTTAGCATTTTTTCTTGGATAATCTTTGATATGCTACTTTGATTAATAATATCTATAAAAATTTTAAATAAAAAGTAACTACATGTTACGATAGTTTGTTTAATGATAAATTTAGTAACATCAATTTTATACGATTGATCTTTCTACTTTTCTTGTTATTAATAGTAAAAATTAAAAATGTTTGACTTAATAGTGTTTCTATTTTAGGACGGAAAGAGTACTTATTGATGATCACAATACTAACAGAGCCTTTTATGTATTTAGTGATTTAATGTTTGATCCCTTCAAAGCTTTCACAAAGTTGTCCTTTCCACACGTCCTCCTATATGCACTTATATAGCATTGCATTGGCACCAAAACTATCGACCTC

>OsGLP3-8

ATGTTTGTGAAGTGATATATCACTATTTTTTTATTTTTTATGATTTTTTAAGTGACATGAAAAAAGAATAGGATATCCCCTTAAGGGATGAGTAGAGTTTTCATGCTATACAAGAGGTGATGAAAGGATTTTTTTTTTCGAGAATGAAAGAAAAATATTTGAAGGGTTAAAAAAAAAAAGTCTCTGACTCACTGTTAACGCAGTATTAATCACTAGTGACTACTCATGGTTACCGTAAAAAAACGACTACGCAGAGTGAGAGATTGACAGGCCCTTTCGGTGGTAATTCCGTGTATAATCATTATCCCGTTGCATCAATTACTTATCTTTTAGATAGTTGTTGAGAACTCTAACTCTCCAAATCGTGATTGGTGAAACGTGTGAGAAACTATTTTGTAAGAGGCAAAGTTCTCAACTATGGCCATTTCACCGATCTGGAAAGTGGCAGTACGCGTTGGATGATCCTAGTAATCGGATTATGATTCATAAGGTCGTAGTTAGAAATTGAAGGTCTTGGCACGAAACATATAGCGGGAGTCTAAAATTTTAAAATATAGTATATCGTAGAGATTATTGAATATTGTTGCGATGACTTATAATCTTATTATGCCCTGGTGGCATGCGATGTGTCAATTTATGGTCTTGGTGATTTGGTGATTGTATGGGGACTACTATTTAACGCACTTGCGTTCCCAACAAATCCCTTTTTTCCCCCTAAAAACTTTCATCCAAAAATTTTTTGTCCGCAAAAGTTTCACCCGGAAAAACATTTATCTATATATTTCATAGAAATACTTTAATTGAAGAAGTTCTATAAAAAACTTTCATATGAAAGTGTGCACTAGAACGTATGTACGACAAATAACATTTTTAGACTACGCGCGGCTCCATGATGAGTGAAGATAGGTAAAGTAACCAGGGCCTCGTTGAGCAATGCGCCAATGCCGGAGAGAGGCGTCGGCGTAGAGGTCTATGACAACAGCTCATTCTCGACCGAAACTGACGTCGCCGCAGTGGTGCAGCCGTGCAGCGCGTTATGGTGGAGGAAGAAGGGCAAGTTGTGGGCTCAGGAGCAGTACATGGATCAGCTTGTGGAACTCACTCGATGAGCAAATCACCATAGTTGAAAACTTTGCCTCTCAATATAGTTCCTCTCATATTTCACCAACCACAATTTGAAGAGTTAGTTCTTAACACAGCTCTCTAAAAGATAGCAACTAATGCAGCGAAATGATGGTTAATTATAGACGGAATTACCACCCAAAGATTTGTCAATCTCTCAAGCTGAGTAGTCATTTTTTGCACGGTAAGTCAGAGTACTCATCTCATCATCTGTAATCAGTAGTACTGCACTAACAGAGAGGACTTTTAAAGTTTAAACCCTTTACCCTTCAAAGATTCAGACAGTTCAAAGTCCTCCAAATATTTATCCTTCCTCACGTCCTATATAACATTGCATTTGCACCACACCACTCGCCACCAATGGAGCACACCGGAGCC

>OsGLP4-1

TACAAGGATGCATTTTTGAAGCGTTGAGCGAATAATCCATTTGCATAATCCAGCGAATATGTTGTTGTATGTATCGGACATTGACAATATTATCTGTAGAATATAGCTTTAATCTCCAATTTTTATTTAAAAAATATGTTATATTTGAAAAGATAATGAAATTTGTGATCGTGTTTGGATTCAACCCGAGTTCAAATCCTGGTTACAAATAGAAACATTTACGCTTAGAATCGACCTATAGAGACAGGGTCCTCGTGTATTAGACACAGGTTGCCTGTTTGGTAGAGCTCTAACTCCTAAATTTAGCTCCAGGAGTTGGGTCTAGAATGGAGTTGTGGAGCTGCCTAAACCCAGCTCCACCTCTCTAGTTCATTCTATGAGAGAGCTCCACCTAACTCTGCTTCTATTTTAGGTGGAGCTGGAGTTGAAGCTGTGCCAAATAGGCCCTTAATTAAGGCACGATTCATAGGGGATAGCTCCTGTCATCCTGTGCAGGGGACCTGCTTCTATGGCCTTTCTTGAATCCAAATTTAGGTATTTCTTATTTGAAATACCTTGGGTAGGTATTTAATTTCCCTTAAAGCCGAGGGTTTCTTTAAAAAAAGAAATGAAATTTCGATATTTTGATAGTATTGTTTTTCAATAGAATTTTTTTTTCATGTTTTCAATCTACTCACTCCTATTCAGAGAAGTACTGACAGCAATGTTTAACCCCACCTAACCCAAAATCACTAATTTTTTGTACTCAATGGAGTATCTTGCTAGATTTTCCAATTAAGCACATAATCGTACATGCATGCATGTACATTCCACAAAGGATGTATACATGCATACTCCAGGAGTATTAGTGATGTCATCCGCAGGGATATGGAACTGTCACTTCTTCAGAGACTTCAGTGAAAAGTAACACTTGTTTAAGAACAGAGATATCCTATATCCAACGTCGCCGCAGGTAGTGGAGTTTACAGAAATCTTTTAACTTGCTAGTGGACGCACGACGCATGTTGGGGCGAAACTGCGTATGAAAATTTTCGTTATCTTCCCCAAATTTTCCACCCATATCGTGCAGGTATTCCACAGACCAAGGATACATATATTATGCATATTTATGCACGTGATTACACTGCATAAACAAACAGTTAGTGGGACTGTAACAAGACAATCAAGCAAAAATAGGTTGTGTCTTACGTAAGAGTAATAATTAACACAGACACAGCTAGCTCGTATTGTGGTTTAATTGTGGATTCCTACTCAAACTACACACAATGAACAGATTAATTTGGAGTAGTAGTGGAATGCAGTGAATATAATCATGTACTACAGCAGTATATTATGCAAGTAGTCCACTTCTTCAGGGTTCAGGGGTTGCCTTTTACGCCTGTATAAATTGGTACTCTTGCGTAGTTTACAAGCAGCTCAAAACACTTAGCTAGCTAGCTCACAAAAGCATCAGAGTTAACGTACACTCATCTAGCTAGGGAAGAAGAAGCTAACTAAGCC

>OsGLP5-1

AACTTTTGTTTCAGTTCTCATATTTTCTATAATTTGTCTGGAGGTCAAAAATTTACAATAAAATCCCCCATCCCCCTTCTCTCCCTTATCCTCACGCCACTCCTCTCCTCACGCTGGCGACCCGCCCGACGCCTCTCCTCCCCTAACCCCTGGCCAATGCCTGCCATCTCCTCCCCCGACCAGCGCCTCTCCTCCCCCTGCCAGTCCCCACGTTGTTGGCAGCAGAGGACATTGAGGCGGGTCTTGGTGGCAGCATCCATGGAGTGGCCTCAGCGGTGGGCGTGGATCAAGGATAGCGTAGCCATCGAGCTCCCAGTGGAGCGGATCGAGGCTGGCCTCATCGGTGAGCGCAAATCAAGGCTAGGGACGGCTAGCGAGCAAGCCGATGTTGGCTTCCCCAACCCCTCTGTTGGGTTTCCCTCCCCTTCCCATGACAACCTCGAGGCTAGTCATGGAGCTTAGGCGGCAACTAGATCAGGGCGTCGCGGCCATGCTATCCTGCTTGTTATGAGTGTCCTCCGCCGACAGCCTCCAGATCAAGGCGGCGGTTGTCGAGCATTCCGGAGGAGTGGGAGATGGCGACGAGCCCCGCGATGGCACAGAGCAGGAGCTGCGTCCTTCTTGGCATGGAGAGAGGGACGACAGCAAGCTCCTCAGCATGACACGGAAAGGGGACACGAAAGATGGGGGATGGTGGGCTCCCCTCCATCCGCTCCGTCCGCCGAGACGAGATCCCTTTCCTCTGTCACCTTTCTCCCCTCCGTCTCTTTCTCCCTCTCCGTTTCTAGGCGACGCAGGCAACGACGACATGCACGAAACCACACACTCTCTCTCCCCATGCCACCAATCTTCGAACGGGCTGAAGAACGGGTGACCATCTTTGGTCACGCGTTCCTCAACTTGGTGGGCCCACCCACTAACGTGGCACCAAATCACACAGAAAAGCTAGCCGACGTGGAGAGCTTGGGCCGAATGACAGCACGATATACAGTGAACGCCCTAAGAAGGGTCATCGTGACCTTTCTAAGATCTTGCGTCAACTTCTGGCTTACAAAAGCAGAAAGGCATCACTCACAATAATTCTTTAGGGCCCGATGTAGCACACTTCCATACCCTTTGCCCCTGTACCAAACAAATCTGCCCACGGGACGAAATCAAGAGATGCACAATAAAATAACCACCCATCGCATTGTTCCTTGTGTACTCCTTGAGTAACCCCCTCCTACTCCCAGCCAGCACATTTCTTGGGGGTGAAAGGACGCAATCCTAGCTAGCTGCTGCTACATTTGCCACTGCCACTGTGCGCCCAAGTCCCCACCTGTTATAAATACTACCATATCTTCGGTCCATTCAGCATCCAACAAAGTCATTCCCTCCGCCATTAATAACAGCCAGAAAGAGTGTAGTAAAGGTGTGTGCACAAAGCAGCAGTAAAGAGCACAGGCAGGCTGGAACAAGCAGGAGAGCGATCGATCCAGTAGGCAGGCAGTGGCAATAGCA

>OsGLP8-1

TTAAAGGAGAATTGGCTGTGTTCATGGAAGGCTAAGAACTGGGTCGATCTAATTTCAATTAACTAATGTCAAGATAGTCAAGATTAATACTATAACACACGCCTGACCATGTGTCAGAATATGTATATTAATTGTAGAATATCGTGAGCTATATATATACGCACGTGCGTGTACATGTAATTGCTGCTTATCATGGTATATGTCAATATCTGATTCCTCTATATATGTACAGGTACAAGAACTTGACAACATTCGTAGAATTAGTTAAGTATTAACCGGTCTCTGTCTCACTGAGCAGGCAAATTTTTTATTGAAATTAACGCTAAATTAAAGAGGCAAACGAAACGAGAAATATGGGATCAAGTGCTATAGTTAGTTATTATGAGGTGCAAAAATTAATGATTCTGTGTTCTTTTGTTCATGAAACCAGATTACATTAGTATCTTTATCTCGCAAATCCTGTGACTTTAATTAATCATTCTACCCTGTTCACAGAGGACGAAGAAATATATATCCCTACTAATTAATTAGACTGGGAGGCCTGTTCTTGTTCTTTAATTTAGATAACAGATCATTTGATCTCTAAAGCCGGCCTGTATATGTTTCTCGTTTATTGATTTAGTCAGAGCATAGGACTTGGATTAGAACCAGAGAGCTTGGTCAGAAAAGATGATTAGATGAATACAAATAGTATACGATAGCAGCATTTCGACAAGAAACATTAATTAGATTAATACAAATATATGGTGGCAGTAATTGGATATGAGTGTTTGGACATGTGTTGACCCAGAATTTGATGTCTCACCAAACAGAACGAGAACAAGATATTCGATCGAATTGTCATTTGTTGGAAGCGAATTAATGGTAGGTTATGCATATGAAAACTATTTGAAATCTTCCACACCTTGATAGACTTGGCATAAAAAATATTTTCGATTCAAAAATTACATACTGGATGTGGATGATTTGCTTCATTAGAAATAAGCTCGTAATTTGTATGGGTTGTAAATTGATTTCATGTAATTTCGTAAATTTTTGTATGTTAGAATAATTTCTTGTTCAATCAATTGGAAGTGTTAATCATTGTACCATTATCAAAGGGATGAATAATGTGACTAAATTTTTAGGGGCCTGATTATGTACAAATGTTAATTAATTTGCTATTACTAAGTCATCCAATATATACGTATGTGTAAATATATACAGTCAAACATGAAGTTAGTGAATAGATAATCAGATATCCAGATATACGTTTTCCAAGAAAACATGAAAACCTGATACAACATACTTAATTAACACGTTATAGACAGCGTCTTCAAAACCATTTCAATCAGTTATGTTGGACGGTTTCACATGACTTGTTCTTGATCTGTTGCACACACAAGGTCAGACCTTGCAGGATCTCCCTCTATCGACGGCTATATAAACACATCCATCCCTTATGTCCAAAGCATCACTACACAAAACAGGAAACACAAAGCATCTGATCAGGAAAACATA

>OsGLP8-2

TGATTTAATTTGATGGGATGCGTACATATTTTGATTCCTTGTCCTAAAGTATGCAAAAATCCCTGTCCATCAGGTGTGTTGTCTACACACGGCTATGTCTCATTGTGTTATATATGTTGACTTGAACTTTTTCGCAAAATGGATTTCATTAATTGGTTCCTTTTCAAAGTGACTTTAGTATATTATAGGAAACGGTGAAGATGACCTCTATACCACCTAATTTAATCGACCTTGTGTTGTTAGGTGGCACATCAAATATCATTATCTATATCTCTACCTATACCTTATATAAGTAACCCAGGGGAAAAAAATCGAACCCATGAATTGTGAGATCACAATTCAGAGATTAAAACAAGGTATGCCAAATATGAGTATATAGTATACCATATAAAATAACTCAAATTCGAATTAAGAATAAACATGAAAAATAGCAATTGGCTTTGAAGATTAATTACGTACTCTGCTGAAAAAAAAACCAAAAGAATCTGGAAAGAACATAAGTGTGAAATTTCAGTATCTTCTCAACAGTACAGAAGAATTATTTATATTAAAAATTGCATCATTTTTTTGGAAAAGGGATATATATATATACACACACACACAAACACACACACACACACACACACACACATTCAGACAGAACATAACCATATAGCCATGCACCCGACCGATGCTAACGGCTCACACTCGCCAAAGTATGGCTAGCTAAATTTTGATCCCATGAATTTTCTATACTCTAGCAGGCCTATCTTCAGCCAACATCTTTTTAATTTCTTCCCTAACCAGAAATTGGTCATCTAAGGAGTCAATTTTTATTTTCTCTAAGTTCAAACAAACTTATTTTTTTTGGGGCGAATGTACATCTAACAGGACCCACAGGTAGACGTGATTTTTTCTAAAAAAAGATGTTATAAAATTGCACCTTGTATCAAAATACTTTGACATATATACATTCCAAAGGGAGAATATGTTGCTAGACACTTGTAATAATTGATTGGTTCAGAAATTAATCACTAATTGTCCGTAAAGGGTTTAATTAATCGTTAGTGGTTACAGTTGGATGATATATGCCAAAATGAACGGTGAATTTCGAATCTTTCTTGCATCTGGTGGCTATTAATTACTTTAGGAGTAAATTTAAAAAACTATATGTATGTTAATATCAAACTATCACAAACTACTTATTTGAGACATTGTATTATAAACTATAGATTTCGCACCAAAAATATCACAAAACTACATATTTAAAGCCCAAACTCAAAAAACTATGGTTTTGTTATATAAACGTTATATGTAAATATGTCAACCAAACGTCGTCACATGGAGAAACCAGATAAAACAGACTGACAGTCTGGAGAACCATTAAAATCTTACAAGATCACACACTGCAAACTGCATGCTCTCTCTCCCTCTCAACGCCTATATAAGCACATCCATCCCCCCTATGATCAAAGCATCACAGAAACCATAAACACACAGGCATCTGATTAGAGAAATCTA

>OsGLP8-3

ATATGAAATTACTTAATCTCTAATTCAAAATATAACCACATGAAATGATAGCCATAAGTTAAATAGTACAGAGAGATGCAGTTTTTTTATTTTTATTTTATTTTTTTGATATTTTTTTCACACTTAGGAGAATACAGGAACCAACCATAGAAAAAATAAACTTAAAACGGATATAATATGTGACTTGTAGAATTTTCCAAAGCTCTACCGAAAACAGACAAAATATGTCATTTATTAAAATAGGCATAACTTTCTCATACGGACTCGGAATCAGGCAAATAATATATCCACGGACATCTACAGAAAAAGTTACATCCGATTCTTCCCGGCTTTGCCAGGTTCGGCGATATTAGAACCTCCAAATTTTGCTTGCAAGATTGTGTTTTCGAGGAGAGAAGTTTTTTGGTAGAGCTTTGGAAAATTCCACAGGCCACATATTTTGTTCGTTTGAGTTTATTTTTTATATGGTCAGTTCTTGTGAGTTCCTAAGTGTGAAAAAAATATCAAAAAATAAAATAAAAATAAAAAAGTTGCACATCTCTCTCCTCTGCACTAGTTCGGAGAGAGAGGAGAGGAGAGGAAAAATTCTACAGGTCACATATTACGTCCATTTGAGTTCATTTTTTCTATGGTTGGTTCTTGTGTGTCTCTAAGCGTGAAAAAAATATCAAACAAATAAAACAAAAATAAAAAATTTCGGGGGGGGGGGGCGCCAGCCACTCTGGCGCGCTCCCCCTAGCCACCTCAGCATCGCCCCGCCACGTCGGCAGGGGGACGGCGCCAGAGAGGCTGGCGCCGTCCAAAAAGGACCATTTCTAGGATAAGTTTTTTCAGGGATCTATTTACGAAATAAGTTTTTTAAAAGGACCAAAATGTGAAAAATCCAGTTATTTTTTGTTTGAGGATGTCGTATCTCTATTAAACTTGCGACCAAAGACCTCCCAAAAATGGGATAGCTTCTTTCCATCTCACCTTCGTCCATAGCTAGAACCAAACAATAATTAGCGACCAATAATAAGAAATGAACAACGAAAATGAAAATGGAAATCACAAAACTACCTAATGAAACATGCCGAGCCGTAACTACATGTATCTAGTAAGCATAAGGTAGATTAGTATTTGAAACAAACAGAGCTGACTGCAGGGAACTAGGAAGGATAATATATATATATTGGTTACTTAGTTACTAGAACAGACATGTTTTTTCAGAGGAAACTTGTTCAAACTGATAAACATGGATGAAATATTTAATGTGCAAGCAGGTCTAGCTAGCAGCCAGAGTACTTTTCGATCACTTTTGGTCAGCTCCACGTATATGTACTTGACTTAATCTGTTTGTACGCAACCAATTATTTTTCTGTCGCCACGCAACACTGCCTCTCTTCCTCTCGATAGACCTCTCCATGCGGCTATAAAATCACATGCATCCCCTCAATTGCAAAATCATCTCTCACCCAAAATAACGATAAACACAGGGAGAGAGAGAGAGAAAGAAATTCA

>OsGLP8-4

GGGAAATTATATACTCCTATATGTGTAACCAACAAAGCAAGTGATCAATGTTGGAAAACGTTAGAGCTAGTGATCAATTAATCAGAAAATGTTCATGACATATTGCATATACCTGCAGCGATTGGTTGGGTTAGGTGCAGGCACAAGCAAGATATAATTAGAATTAATCTCAATGACGTGGTCAACTATTATGCGTATATGCATGGATATGCATATGCATAAATGTGCACGCATTGAAGTGATGGGTGCCCTTCATTTCTTCATATATAACATCATACAAAAAACAAAATCCTCATATTTCTACATATATATTATCGTCATTGAGTCCATTTTCCGAAAAGAAAATGATCAATTCAGTGATCCATATGTAGGTCTTTTGAGAATTCACTAAATTAATACTCCCTCTGGTTCTATATTAATTGACGTTTTAGACAAGGTTGAGGTTAAACTTTTATAACTTTGACCCTCAATAACTTTAAAAATATTTAGTTTAAAGAAACTAGAAAAAACATATATAGATTTGTCTTTCAAAACACTATAATAAAAGTAAACATGCATTTATTTATTGTATATATTTTAATAGAAAAATAAGGTCAAAGGTATATCTTGTAGAGCATGTCATTGTCCAAAACGTCAATTAAAATAAAACCGGAAGGAGTAATAGGGACAATATTGGGTACGTGGAGCACGTACCTCCATCCTAATTTAAGTGTGGTTGTGGGTTTTCGTGTCTAACGTTTAACCGTATGTCTTATTTAAAAATTTATAAAAATAAAAAAATAGTCACACATAAAGTACTATTTATTCTTTATCAACGAATAATAACAAAAATAATAATCATATTGTTTTTAAATAAGATGAACTGTCAAACGTTGGATATAAACAGTACAAAACTACACTCTTTCTGGGATGGAAGCAGTATTCTAATTGATGCATGCTAGTTTGTTGGAGAAAGAACAGACATTAGTTTTTATATGGACGTCTTTGACAGGTCTTTTCAGTGCCTTCACTAAATAATACTCCGGATAATGGATCGATATGACGACGACCTTTTCTGTACCAGCCAGATGCAGCATGCAATATTGTAATCCAACTATAGTTTTGATGTGCATGGCACAAGATATACATAAATCTAAATTGCTGTTCAGTGTATACGTTGTCCACGGTCAAGCACGATGCAAATTAGTTATTGGAGAGATATGTATATTTAAACAGAAACCACTTAATTAAAACTGAGACGAAATATTTAATCATCAGCAATTAATTAAAGCACTAGCAGCGAGTACTTTTCAATCGGTGAGTTCGCGTGGTCAGCTACACGTACTTGACTTGTTCTGTTACTCACAGCTAATTAAGCAATTTAATTTTGTCACCACAATTTGCGCTCCTTTAATTTGAACCAACCTTTCCGCTATAAAATCACATCCGATGCCTCCGTCGCCAAAGCATCTCACACACAGCAGGAGAAACCAACTCATAGTAGCTTAGCTAATTAACACG

>OsGLP8-5

CACTCATAGAGAATCCCTAATGCATAATTTAGTAGCCAAAATTTGCACACAAAATTCTACCTTATGCAACTAAAGGATTTCCATCATGTCATTGGTGTTGGCATGAGGAGCTAGCCTGCTTGTGACAAAGCTAGCACTTTGCAGCTAGCCAAGCAGTGAGCATGCACCTACTATTGTTAGGGATAATTATATCAAGGTGTGCTAGATGTTTGCATTATTGTATACCAGACTAATAGAATGGTCGGTTAAAATTAAAGATTACCTTTACAGAAAAAGCAATGGTCTGATATTTAATTTTGAAAGAAATCAACAATATATATTCCATTTTTTTAGAAAATGGATTAAAACCTGGCCTCTACACCCAAAAGTAGAGAGTTTTTCTTAAAAAAGGTGTTAGCAAGTCTAACACAATAATATATTTCTGTATGTAATTTACATGTGTGTGAATATGTGATGGCCATTTATGTTGTGAACAAGTCAATTAAGCTATAACTAGCTAGCTCGAAATATAGATTGTTGAACTTGTCTGGACGTACTAGCCGCTCACAAGAAAGCGTTTTTTTTTTTTTTGTAATCCAGATCGTGCCAGTTCTCCTATTATTTGCCATCCGGATTTATATACATTGCATCCAATGTTTCGTGAGGAATAACAATTAGATTTGGTGCAATGACAATGGTGAGACCAAGTCTCTACTATTGTAAAAATTAAAGATGTTTTTGTCGGTACTTGGTATACCATCCGTGTATGAGTCGGTATTTAAATTCATTCGCTTTTGGAAATACAAATTCGTAGTTGAGTTGATTTTTAAATTCGTTCACTTTTACAAATACAGAAGGAATCGTAGAAGGAATCGTATAAGAAATCTCTTTCAAAAACTAGCACATTAATTTGAGACGAAGCTCATGATTTTCTAACAAAATATATATCCAAGCGAATTTAACATAGTGAATTTCATCTTAACTAAACCATATAACAATAATAAGATTAAAATAGCTTCATCAGTTACAACGCACGGGTATTTTTTCTAGTAATTAAAAGGATGGAAACGATGGTTTGAGTACTGACTTGGCAATGCTGGTCATTGATTAAAGATGTACACATATTGCCCCCTTTAATTTGCAGAACACACAATAACCACTACGTCTTCTTTTTTGAAAAAAAAACAAAAAAACCCTACTATGGCATTCTCAAATGTATAAACACAATTTTGACCACGTAGTTAGTAGAGCAGGAAGATATTTTAAGCTGAAGAGTTCAACCTGTTGCCTGCAGGCTGCAGACCATTTCAATCTAGCTGACATCATCACTTCATCTTATTTGACTTGTTCTGTTCTTCACAATTAAGTTCACCAAAGCCACACCCTGCAGCTATATAAGCACATCAAAACTCCATTGCTAAACCCATCACCCAACAAAGCAGTAACTAATTCTAGCTAGCTCAGCTAGAGAAACAGCAAATAATTCAGCAAGAGGAAGAGCAGAAATTAATCCAAAGCCGA

>OsGLP8-6

GTTATCGTATCTTAGAACTTTGAAACAAAGTTTATATATAACTTTTGAAATAATTTAAAAATAATGTATTCAAATAAATATGTACTACCTCCGTCACAGAATATAATAATCTAGGACTGGATTAGACATATCCTAGTCAAACAAATCTAGACAGCCCTTATCCAGATTCATTCAAGGATATGTCCCATCCAGTCCCAAATTGCTATATTTTGGGATGGAGGAAGTAAATTTTAATATTTATAATATTAATAATACTACATTCACCTCCGTTTTAGGTTATAAGACTTTCTAGCATTGTTCATATTCATATAGATACTAATGAATCTAGGCACACATATATATCTAGATTTATTAACATATATATAAATGTGGGTAATGCGAGAAAGTCTTATAATATGAAACGGAGGAAGTATTTGCCTGTGTAATATAAACATGGGTTGGCGCCTAGTTTATGTAAATATACTAGCGAGATGAGCGCTCATATGTACCAGGCTTGAAAACAATAAAAAATTAACCTCCACTAACTTATAACCTTCACATAGAAAACATATGGGTCTTACGTTTTGAACAAAATTAACAATATCGATTAATTCTGTATATGCAGATATGTTTTTAAGAAACAATTAATTGCTATCTACTTTGGAACAATTAATAATTAATATATAATGCTGTATATTTTTACATGTGATGGTTCTTTTATGAACAAGTCAATAAAATAGCAAAGTAGCAATGTAAGGTTATTGAACTTGTCCTGACTAACCGCTCAAAAGAAAGCGTTCAATTTTCTAAGCCAGATTGTGACAGTAAACCTCCTAATAATTTTTGTCATGCATGACAATTAACCAAGCTTTCGTGAGGGGTGACAATAATTTTATGTCGACGGCTAGAAAATATTAACCAAAGTGATTAAAAAGGTGGCCGAGTTTAGTTCCAAAATTTTTCTTCAAACTTCCAACTTTTTCATCACATCAAAACTTTCCTATACACACAAATTTCCAACTTTTCATCACATCATTCCAATTTCAACCAAACTTTTAATTTTGGCGTAAACTGAACACACCCTGTATCTACACCATCGATCACTTCAGATTAGTTAGGGTGTGTTTAGTTCACTCCAAAATTGAAAGTTTGGTTGAAATTGGAACGATGTGACAGAAAAGTTGAAAGTTTATGTGTGTAGGAAAGTTTTGATGTGATGTAAAAGTTGAAAGTTTGAAGAAAAACTTTGGATCTCAACTCGGCCTTAATTAGTAGCGCAGATACATACTTTTGAGCTGAAAAGTTCAACCTGCAACCTTCAGAAACCTGCAGGCTGCAGAACATTTCAGTCGCTGACATCAGTTCAGCCTGACTTGTTCCAATATTCACAAGTTCACGCTGCCGCGTGCCCCGGCTATATAAACACATCAATCCTCCATAGCCAAACCATCACCCCAAGAGAAACACAAGCAATATTAGTTAGCTTCCCATCAGAGAAAGATAGCAGAAATCCAAAGCCAA

>OsGLP8-7

ACATCCCATTGCTCCATTGGACATGGGTGATGGCAAAGGCCGATAAAGAAGAGGTGACAAGGGGAGCACAAGGAGAGAGGGTGGCAACAAAGCCCCACAAAATAAAGAGTGCCACCGGTTGCTTCCAAGATAGTAAGTAGTATGTAAGATGTCATCTAAAATCCCTAATGTATGAATAACTAAAATTTGCACACTAATTCCACCCTCTATAATTATAAAGGTGTTCCTTCATGCCGGGGTGGTACAGTAGCACTTTTCATGTGGTTGGGTACATACACCGTTGTTAGGGTTTATTAGCAAGCTGCATGAGAGAACTGTTTGTATATATAGTACTACATACATAAAACAGTGGTCGATTTACCTGCATGGATGGATATATAACTTTTACACAGAAAAAATATATAAGGGTACGGTGTTATTTTTAAACGAAATCAACAATTAATATATATTCCATAATCCATATATATGCAGATTTTTTTAAAAAAAATAGCTAGCTAATGTGGCATAATAACATATTGCTACACAATGTTACAAGTAATAGTTATATCGTGAACAAATCTTGACTAAACGCTCACAAGAAAGCGTTTTCTTAAGCCAGATTGCGACGGTTCTCTTAATATTGTTTGTCATGCATGCGTGGCAACCACATGGTTACGTGAGCGGTGACAGTCACTAATTCACTACTCCCTCTACCTCCGTTTCAGGTTACAAGACGTTTTGACTTTAGTCAAAGTCAAACTACTTTAAGTTTGACTAGTTCTGTAGAAAAAAAGTAGTAATATTTACAACACCAATATAGTTTCTTTAAATCTATAATTGAATAAATTTTCATAATATATTTATCTTGGGTTAAAAATATTACTACTTTTTTCTATAAAATTAGTCAAACTTAAAATAGTTTGATTTTGAGCAAAAACAAAATATCTTATAACCTGAAACGGAGGGAGTACAAGAAAAACGATTTCGCAGGCAACCAAATGCATCTACGTAGGCGGCCAGTGCCCAGAGGCCTTTGTAAGGTGACCAAGTAATCAAAAAGGTTGGAAATGATATATAGTGTGTCATTGATCAATCGATGACGCACACAAATTGGACCTTTCAATAATGTGCTGATCAGATCATAACAACTACAGTGACTTCTTCCAAATATACGTGGACTATCGTCCATCGGGATGCTATTACGCGTCGTCGTCAAAATGTGTGTAAACACGATCGACCACGTCTGCAGACATATAATTTCTAAGCTGAAAAGTTCAACCTGCAGGCAGCAAATTGCAGACCATTTCAGTCTACTAGCTGACACCAGTCCAGCTCAATATTGACTTGTTCAGTTGTTCACAAGTTCACGCTGCCGCGCCACTACGGCTATATAAGCACATCAATCCTCCACAGCCAAAGCATCACCCAACGATAAACACAACCTGTTAGCTAGCTAGCTTCTCAGCCACCAGAGAAACAGCAAATAATTCAGTAGAGAAGAGAACAGAAAACCAAAGCCAA

>OsGLP8-8

GGGTTGTAAGCGGCGAGGTGGGGGAAAGAGCTAGTGGCCCCGGGCCTATCGGATCTACTGTGCATCCCCTCTTCCTCTCTGCTCTCCTCACATCAAATCTAAGCGGCAAACTTGCTGCTTTGCTCCCGCTAGATTTAGGAGACACCGATCTCACTTCTCAGGGCATGGAAGGGAGTGTTGCGTGACATGGAAGAGAAGTGAGAATCGGTGGGGAAGGAGAAGGTGTGTCCTTTGAGAGAGAAGCCAAATAATAGGGCTGCCAAAAAGGCTCAAGGCTCACGAGCCACTCAAGCTCGGCTCGAGCTTCAAACGAGCCGAGCCTGAGCCGAAACTAAAGCCCGTGAGCTTCTAACGGGTCTAACTGCATATGAATTTTATTGTTTATTCGATACACAATGAATCAGCTAGCATGTATTAGCAAAATAGTTTCTCCTCTTCTATTAGTGTGATTTGCTAGAAAATAGTTATTTCTATTAAATGGTATCAAGTGAGCTAGTAACTTTCAGTGCTAAAAAACCAAAAGCGTGAACTTTCGATGCTTTAAAATCATTTTTGCCTTAAACGTTATACTATTTATGTCCGAGCTCGAACCGGGTTGGCTCAAAAGCTCAAAAGCAGAATAGTCTCAGCTCATCATGCTTGTGTCAAGCTCGAGCCTGGGCAGACAAGCTTGGCTCATTCATAGCACGTGCTGCCGATAAGGGGCTTGCCGTGGGCTTGGGATTTTGTTTGGCTCAGCTCGGTTCACTCCCTGTTCATGTTAAAAAATCACAGTGATAACATTGTCGGTTTGTAATATATCCCTATCATTTTATGGTGGGTTCAGGGAGAGAACCCATCCAACCACCAATCCAACATGAACCGCCCAGCCAGCTCGAGTAGAACCGGTAGTGATGAGCCGATAGGGATTGCATGCCCTTTCTATTATAGTCAGTCAACACCTAACCATGGATTGCTCTCAGATTTCATCCGATGTTTCGTGAGGGGTGACAAATTAATTGATTTGATCCCAAGATAATGGTGACCAAGTAATTGAAAAGGTTGGAAACGATAGTGCAAAGACTAAGCACGCAGGCTGTTGATTGATGATGCACGCTGATTGACCGTTCTATGCAGATCCCATCAAAAAGAGAGAGAGAAAAAACTACTCTACGTCATCGTCAAAATGTGTATAAACACAATCGACCACGTCAGATTAGTAAGTAGGGCCAAAACGTATTTTTAAGCTGAAAAGTTCAACCTGCAGGCAACAAATTGCAGACCATTTCACTCTACTATAGCTGACATCAGTTCAGCTTAATTGACTTGTTCTCCATCACAAGTTTACGCTGCCGCACCCTGTGGCTATATAAACACATCAATCCTCCGTAGCCAAAGCATCACCCAACGATAAACACAAGCAGTTAGCTAGAAGCTAGCTAATTAACCTTCTCAGCCACCAGAGAAAGAACAAATAATTCAGTGCAAAAGAGAGCAGAAAAAAAGTAACCACAAAGCCAA

>OsGLP8-9

GTGCTAGTTTATTTAAAAAAACAGTACTAATCATAAAAGTGTTGGTTTTTTTAATACAGCAACACCTATACGTAGGATAAAAGTGTTGGTTTTATTAAGGAATAAAAAGGACACTTCAATGGTGAGCCTGAGTGCATGTGGAGTATGGAGTGTGGGATAAGTCCCACACTTATCCTTATCTTCTTCGTCTCTCCCATCTCTCATTCCCTCTGCACTCAATCTCTCCCGTCTCCATCTCTCCTGTCCCTCTGCTCGGTCGGCGAGTGGCAATTGGACGCCTCTCATGGGCACGGTGGCCACCAGCATAGAGGCGCAGCGGCGCCGCGTTTCTGTGTTTTGTCGATGCAACAGTGTAGAGGTGGTCGCATCTTCCTCCACCATCATGCTCGCCCCTCCCTCTCCTCTCCTCTCCTCTCCTCTCCTATCATGTCCCGTGATGTGGACGAGGAGGCACGGGGACCAGATCCAGTGGCCCCCGAACTTAGGACCTCCACGGTGGTGAGGAGCAACAGTGGCCTTCTCTTCCTCTCCATAATGCTGCAGCCACCGGATGCGGCAAGAGAGAGCTTAATCTGGCTCTCCCGCTCATCCCACCCTCTCCTCTCCTACGTTTTAAACAGGAAGTGATAACTAGGCATGCCTATTTACAGCACACAATATAATTGTTGCAATATGTTTTGTTGTCATCAACAACAAATTGATCACCCAGTCATCAAGGATTCGTACAAATCACCAACAATCACATCAACATCAATCTAAATCACCAACAATCACAACAACATCAACCTAGATATGACGTCGACATCACAGCAACATCACAGAGAGAGGAGGAAGAGGTGATCGATCTAGATCGACTTAGCTCATCGCCGTAGCTCTACTTTTATCGTACCCAGTGGTTTAATTAATTAAGCAGAGACACCCATCCAACCACCATGAACCAGCAGCGATGCTTAATCTCTAATGCAAATTTGAATTGATCTCAGATTGCATCCGATGTTTCGTGAGAGGTGACAATTTGATTTGGTCCCAAGATAATGGTGACCAAGTAATTGGAAAAGGTCGGAAACGAGAATTCCATGACTTAGCATGCAGATCATTGATTGATGCTAATGCACGCTGATTGGCTCTTCTGTACAGATCCGATCATAACTAATGCCATCGTCAAAATAGCTAGTGTGTGTCATCGTCAAAATGTGTGTAAACAGAATCGACCACGTCAGATTAGTTATTAGTGCTGATACATATTTTTAAGCTGAAAAGATCAACCTGCAAGCAGCAAATTGCAGACCATTTCAGTCTACTAGCTGACATCAGTCCAGCTTAATTGACTTGTTCAGTTGTTCACAAGCTCACGCTGGCGTGCCCAGCGGCTATATAAACACATAAATCCTGCACAGCCAAAGCATCACCCAGCGATAAACACAAGCAGTTAGCCAGCCACAAGCTAAGAGAAACAGCAAATAATTCAGTAGAGAAGATAGCAGAAACCCAAAGCCAAAA

>OsGLP8-10

TAAAATCCTCTAGCTAGAAGAGCGGGTTCCTAGTGCCTTTATTACTCGGTTAAAGACATCTATGTCTATTTGAACATCCGGCAATGATGATCCTCCCTCATCAGTGCCAATTCAGAACTGCCGGTTCCAAATCCAGCATTGTTGGGTCCTTTGAAATATGTGATGTGTTCTGTACTGATGCTAGCAAGGGCATGTCTAAAAGGTAGGAGAAGGTTTTCGACACGGGTGTATGTAATCCTAGTTTCATCATTTCATGGATGGTTTGGAAAGCATGGAATGCCCGCGTCTTCTATGCGTGTTTCGCCTTTGCAGTTTATTAGACCGCGGCAACCATCCAGGACAAGTGGCAAACTTAGCCTGGAAAGAAGCTGGTCTACTTGGCATTGTAGAAACCATTGAAACTTAACCTGCCAACCTATGTGACCTCTTCCTTTTAGAAACTTGGCTTACGATGCTACATCCCTAGTGGATGCTGATGCATACCTCTTACCCTTCTGGTCAGACTGGTTATCCTTCAACATGTTAGCTGAAAAAAGTACTATGTTGCATGCCATCAATCTCAATAAAAGTTTTGATGACTTCCACCGACCCGGCAAAAAAAAAAACAGATATATAGAATTGGTCTTATTAATAACAAGCCTTATTAAGCTAATCGATCGACAGGACTAGCGTTCGGGACAGCCTGCATAAGTGTCCGTACCAGTACGGTCAGAGTGACATATGGGAAAACCAGTACAGTTAAACTGTATTTATTCTCCAGAAAAAAAAATTGTAGTCTTTGTTACGCCTGTAATATTGCCAGTGAAAGTAATTAACCACTCCCAAGAAAAATGCATGGCGTGTGACCAAGTAAATTTTTGACTAGTCTGCAGAGTGATCAGTGACAAAATTAATGATATGCTAAAAGGTAGCAAAAGCTGCTCGTAATGTCCTTCTCATAATCCTTAATTACCGGCCGGTTCGAAACAATTTGATTTGATCCCAAGATAATGGCGACCAAGTAAATGAAAAAGGTTGGAAACAATAGTTCTTTCGATCGATGACTTAGCATGCAGGTCATTGATTGATGATGCACGCTCGTTGGCCCTTTCTGCAGATCCGAACATAACTACTGCTATCCTTAATTTCCTTCAAAAAGAGATAAAAGATAACTACTCTATGCCATCGTCAAAATGTGTGAAAACACAATCGACCACGTCAGGTTAGTTAGTAGTGCCGATACATATATTTTTAAGCTGAAAAGTTCAACCTGCAGGCAGCAAATTGCAGACCATTTCAGTCTACTAGCTGACATCAGTTCAGATTAATTGACTTGTTCAGTTGTTCACAAGTTCACGCTGCCGCGCCCCTGCGGCTATATAAGCACATTAATCCTCCACAGCCAAAGCATCACCCAACGATAAACACAAGCAGTTAGCTAGCAGCTAGCTAGCTTCTCAGCCACCAGAGAAACAGCAAATAATTCAGCAGAGAAGAGAGCAGAAAACCAAAGCCAAGTAA

>OsGLP8-11

AACCACATATCAAATGTTATTCGTTTCTATGTGGGTGCAGTGATTTCAAAAACTTTGTGGTTGTACAATGTACTTTCTATGTTTCTCACGTTGGTTAATTAGTTCAAATTTGCATTGCACTAATCAACATTAAATAAATGAAAAATGCAGGGAGCAATATTAGTGAAGTTTGGACGGCTTCAGCAGTTCCGCTGGACCAACGAAAAGATTGAACAAGTCTTATTACTAATAAATCTTACTAAGCTAATCCACAGTGTGTTAGTGTTTGTCACAGCCTGCAGCATGCGTGTCCATAGGACTCCGTACCAGTAAGTAGCTAGTCTCTATACTGACTACTAGCAAATCCAATATTAGACTATATTTGTTCCCTAGCAAAAGAAAGGATAATAAAAAGGTAGTACTGGTCTTTGTTACGCATGTATTGCTGGTGGAAGTAACCACTCCCAAGAAAACAATGCAGGGTGACCAAGTAAAGTGTTGGAAAAGTATATTTGCAGATTGATCATTCGCAAAAGTGTTGGAGTAGTATTACATGTGATGCTAAAGGGGAAAAATGACCTAAACCTATCGTAGAATTAAAAATATACGTCTCTGAGATTTATCTACGTTACCATTAGTAAAACTTTTATTCTCAGATTTAACAATTAATTAGTAGAAGTGTTACTTATGATAACATGAACAAATCTCGAACATTAATTGTATATTATATAATATCTTCTAGGTTTACACTATATATATATATATGTGTTGTTCATTATATATTTTACACTAGTATTAAATAATGCTAGCTCTTGGTGATATCCTTCCCAAATGATCATCCATCGGCCGGCCCGGTCTCCGTACGGATGGGATTGACATGTGTTTCTGACGTAGGATCAATGTATCTACTCTCATAGATATATACAGGTGCATGCATTGACCGATCTCGATCGACAGTCACTTACCGTTGTCCGTTGCTATCATAGATCAACATCATTACTTGGTTCCTGAGAAATCTCCTCAGTGATTAAGCTATACTTATAATTAATATGTATATATAGTGGACGATGAACGCATGTGTGAATGATCTCGACTGCGTCTTCCCATACAGTTGCAAGTAAATTATACACGGTCAGCGACACACGTCAGATTAGTTAGTAGAGCAGATAAACGTTTTAAAAGAACACACACAAGAGTTGATATATATATATATATACAGATTACAGTGATATATAGTTAATATTAATGAAGTTCAGCACGTTTTTTTCTACCCTAGCAGCCATGCATGCCAGAGACCATTTCAATCCTTTAATTGGTCACAGCTAGCTGACTCTAGCTACACTTGACTTGTTCTTAAATTTAGGTTGCTCGCATGTATGCTTATGCAAGTTCAGACAGCAACATGTTCTGTCTGCTCCTATATAAGCGCATCCTCTCCATGGACAAATGCATCTCAAACACACCACCTGAATTAACAAGCAGGTATATATAAGCTACTAGCTAATTAAGAAGGGCATTAGA

>OsGLP8-12

GACCCAATCTAGTGTAATGATTCTCAATATCAAAATTGTATAACTCGACGAGGTCAACCATTCTAGATTTGATGACTATTCTTTTGATGTCACTAGCAAAATGCCCATGCGTTGCACCGGGTAATGTCGCGTTGGATAAAGTTTAACTGAACGATTTTTTAAGCGGTATAGTATGACAATAATAGTGATCAAGTAATCGTTCATAATTTTCTAGCAATTTTAAAATGGCTCAAAATAATGCCAAGAAAATTTTGTAAACGACTAAATAAATTAAATCGATGGAATTAAATAAAATTCTATTTCGACCTATTACTTTTGTAACTGACCCAAAAAATCGGATCGGCCCGTTTAGCGCGAGCCGATTGCACTACAAGTGGCCCATCTACCGGCGACGGCCCGACACGCGGCAAAGGGCACGCGGCTCAGTTGTCTTCCATGGCCCAAAGACTGCACGGCCCAATAGCGGTGGCGGCCCGATGCGGGGCCGATCTGACCCATCCGATCTGATGGACAGCTTGGATTGGTCCCGCGCCAATGAAATCGCCGGCCGGAAGGGGAGGGTCCGAAAACCCTAACCCTAATTGCCTTTCTTCCCTACTCTCCCTGATCCAATCTCTGGCGACGCGAGAGAGTGGACGGCGATGATCTAGTCTTTCTCCGCGTCTTTCCGTCTTCCACCCGAGTTGTCGCCGACTAGATCATCCACCGGCTCCAAGCCGTCATTCATGCTCGTGTGGATCCGCCGTCGGCGCCTAGATCCCATCGTCTCTCGGCGATAGCCGCATGGCAGCTGCGATGGCCAAGGCGCGTGGGCAGCAGCGAGACTGGTGGGCGGCGGATCTGCCGGCTGGGGAGGCTTGAGGGCGCGGAGGTGGCTGAGTTTTGGCAGCCGCCTCACAAAGTTGCCGCCACCGCCTGCTTCCTCGACATCGCCACTAGCGGGAGATGTTAACAAGGCTAACATGTAACGTATGAGCCCCAAATTTGAATCCTATATGCTACATGTGTGAAAATTTGTGTGTAGATGTATAGTAAAATGTGAACTTTTTTTTATGGTTTTTTCACCTATAAAAATAAAAATTGGAAAATTATTTTCTTGGTTGGTTTCTTAAGAGAGCCGTGTATGAAAATGAGATCATCACAAGAGTTTTGTTAAGAGGACCGCCAAATGGTTCTCTCCTTGCCGCTCGATGCATGTGGAAATAGTTGTTTTTCTACAGGTTACAGCTTACAAGTGACATAACGGTTTGATAAACGACTGTAGCCAAAAGTACTTTTCAATCACTTTAATTTTGGTCAGCTCCCGTACATGTAGTTAACTTGATCTTTGCACGCAAGCAATTATTTTTCTGTCACCACGCTCCTCGACGACCTCTGCATACGGCTATAAAATCACATGCAACCCCTCAATAACCAAAGCATCTTACTCAAAGTCTCAAACGATAACCACAGGGAGAGGAGCTAGTAAAAATAGCTAGCTAACTACCAGAGAGAGATACA

>OsGLP8-13

TTTTATTTTTTCTACTTTCTTCCTTCTTTTTTTTCTCCTTCTGCCACTGGCCCACCTGTCAGTGGGCCTCTCTGACCTCACCTTCCCTTCTTTCCTCTATCCTCTCTCTCTCCGGCCAGTTGAGCGGGACATACGGCCGGCGACCGAAGCGGTGACGGCCGGGGTGGCCGGCGAGAAAGGAGCAGCGCGTGGGCGCCAGCCGACAGCAGGAGAGGAGCGGCACATGTGCACGGGCGGCGGCTGGAGAGGAGAGGAGCGGCGTGTGTGCGCGGCCGATGGCCGGCGAGAAAGGAGCGGCGCGCGGGAGCCGGCCGACGGGGGGACGCGCGTGCACGGGCGGCGACCGGAGAAGAGAGGAGCGGTGCGCACGCGGGGGAGGCGTCACCGACCACGCCCGAGAGGAGAGGTCGCCGACCATGGCCACGTGTGTGGGGGGAGGCGTTGGCCATAGAGCGCGCGAGCTCGACGGCGTTGATGAAGCCGTTGCCGCCGGCGGCCTCCCCGCCCCATTGCCTGGCGCTGTTTTTGCCACCGACCTGCTTTGCCGGCCGGCCGGCCGCCCCCACTCCAACCCGCCCGTCGCCTGCTTCGGTCGTCGGCCGCCTGTCCCGCTCAACCGGCCGAAGAGAGAGAGGAGGGGAAAGTGAGGTCAGAGAGAAGAGGATAGTAGTGATGCCCTGACCCATAGGTCCTACACAATTTTTTTTCATTTCTTTTGTTATTGTAATGCCATATCAGATAATGAAGACTAAGTTAACTCGACACGGTGATGTCTAGCGAAAACCAACTTCAAAACCACCAAAAGAAGTTGTTTTGCACCCGTTTTAGTAGTTGGTAATTAAGATATAGGGTATTGTGGTTGAGTGATACGAAATAGATTGGACCAATATTATTCCTAATACAAAGGAGTTGGCATTGTGCAACCCAGTGACAATCCGCCCCCATTATGTTCAGGCCCAACACAGACAATTCAGGCGAGCCCAATGGGATCACCACCCTAATGATGGCCCAACTTGTGAGCCCCCTTGCCTTTCTCAGCCTTCCACCCTCCAACCCAATCACCAATGGCTCCGCTCCGAAAATCCCCTCAGATTACAGTCGGCCGGTGCCGCTACCGCCGCGGCGACTCGCCGGCTGGAGCGGGCCCCGTGCATCCGCCGTAAAAATGACGAACGCATCAACCACGAAGGGGGAAGATAGCACCTGGAGAAAAAAATCTCTTCATCTTCATTGGCCGGCGTGTGATCGTCGTCATACACTCGTACCCGTCTTGCAAAGTTGCCAATCGTCAAGGCCTCGCACGGACGCGCGCTTCCCTGCACACGCAACCAGCAAAGCGTCGTCAACTTCCCGCACGTTGCTCTGCTCGTACACCACACCTCCACACCTACACGCGCGCGCGCGCATGCATGCGCACGCCCCGTCCTTCCCGTGTATAAATAGCCCAACAATACACCACTGTTCAGTCATCTCATCACCTAGCTACAGTAGTAATCCACC

>OsGLP8-14

CCTTTATTTTTCAAAATTTAATAAAGGTTTTATTTTTAGACTTTTATAATTATTTGAAACTAATAGAAAGTGGCATCAATCAAGTTTTTTTTCTCATTCTATCTCAGAGTATCCATTCATTCGATACTCATTCTCTTTCAGTTGGACTTTCTGAAATTGAGGAATTCAAGCTTTTTCTAGCTGCTCAATGGTCCATTAATTTGTGCAATTCTTTTGAATTTCAAGCTAATGGTAAGATCCTTTTGTCTTCAATTATCTAAGAAAAAAAGTCCATATACCCCTTAAATTTTGACTGAAAATTTATATAGCACCCTAAACTTTCAAACCGGATATTTAACCCCATAAACTTTTCAATACCTTTTATATAATCCCTAATTAACGTGGTTTTGCATAGTTTTCTTTTATCTAGTTTGTACATAATTTGGAAGAGTTTGATCACGTGACATACACATTTCACATATATTTAAATCTTCACTTAAATATTTATTTTTTACTATTTCTTAGCTCCTACTTGCAAACAACTATCAACATAATAATAGTATGATATCACTGTACAGTAAACTGTTGATATGTAAATATATTATATTAGTTATTCAAAGTGACATGTTTTAGCTTTCAAATATGCAAAACCATCATCCAAAATCATCTTAGGGGATAATATGAATGGTAATCTAAGTTTACGACTTTTACAGGGACGTCCCCTGCATGGAAATTTTGTTGCTGGTACGAAGAAATTGATCGAACGCCGGGCCATTTTCACCGGATAGGTTTGGTCTGTTGTAAAGTACAGCTAGCCGCGGGTCCCTATCCATGCCATCGTAAAAATGCCCATCTTGTCTGGGCTTTCCCAAATAATGCAGAAATCCCAGACCTTTTCTTCATCATAGCCCGTCCTCTTCATGGACCACGGTGTACAGCTTGCTTAAAGCTTGTCCATTGCCGGCCTCCTCCCGCTAAACCCTAGCCATCCGCATGCATGCACGTGAACTACTCGATCCACCGCCGCATGCACCACCAGATTCAACCCCATGTTGCTACAAACATGTAAGACTAAATTCAACTTCTACAGGTTAGCAATAAAAAACAAATTAAACTGAAAATAGTTATCGCACATTCGCATATATATTTGTTATTTTTGTTACAACTGGTAGAAGTTGAATTTAAATTTTTATATTTGTGGAGTGATATATTTCATATTAATCTATATTACTATTTTTTAATTTTTTTATAATTATTTAGGTCTCATGCACAAAACAAGAGGATATTACCTCGAAGGATGAAATAACTTTCCCCGTTACACTGTCCAATCCGTTTATTTACACGAGAACGTACGTACGCGCGCTCGATCTCGCGTCTCCTCCCGGCCTATATAAACCCCAGTGGCGTTGCACTGAGCCCTAGCTCAAACACTGTGTACTACGCAACACACACATAGGCACAGTTAGTGCACGTACATCTTGCTGAATTTGCTTGAGCTCGTACGTGCGTAGTGTGGTAGCC

>OsGLP9-1

TCGAGTCGAGTGTGCTAGTATATATTTATGTATGTTTTGACTTGAGTTGTGATCATTGAAAACTTAATTCGATTGAGATATGTCAAAATTGAATTTCTGTTTTTCCCTTTCTTTTTTGAGTCGGCGATTGTTCTATTCTTGTTTCAAGTAGGCATGACCAGCTAGCGCACACTTTTTTCTTTTAGTATCACTTTCCTACGAAACTTTCCACTTAAAACCATGTGACTTTCAACATATATAAATCATACAAAACTTTCAACTGTCGCCACGAACTTTCATTTAAATACATTCATCTCAAACTACAGATTGTATAGTTTAAATCCCTAACAACGATGTCCTGTTGGAGTTCTTAACGTTGTAGTGATGTGATTAAGTTGGCGCTAGTTCTACGATAACTTCATCATATAATATATTTTTGTGATTCTCTATCTTAAATCTATAGTTTCATGATAAATTAATTTGATGTTAAATAAATATATATATATAGTTGTGTGATAGTATTTGTTGTTTTGTGATTTTTTTTAAGAAAAGAATGACTACCTAGTAGTTGTTGCGCACATGCACTTTTTTTTTTTGAAAATAGATTAAAATCCGGCGTCTATATCTCAATGGATATACACAGCCATGCACTTGGTAGCTGCTTAATTACCTCTAATAATTTAATTATTTGCGTTTGGAAACCGATGGATGATCATTCTTCTTGCATGGTGCAATATGCTAATGGAAAAGGCTAAGGTGTAGTGCACACAAGAGAATTAATAAGACCACTAAACCCAAATGCCTTGGAAACTCGATCGATTGTTTTATGTATAGTACACAAATGCAAATGCATATTCTATACAGCAGGAAAGCTAATCAGACAGCAATTAAGACCAAATGTTGTAGTTGTTTCCAAAGCGAGCAAATATATGGCGGCACAAGGCGCCGGCTAATATACTACACATGGCAGCTTGATCAAACTGGTCCAGTAATTAGTATTTCAACAATGTTTGTACACTGGGGGTCTGGACTGTTAGCTCTGTTCCGAATGACGACGTACGCACGCACGTATGCATATGCTCTCATATATGAATGTAGTAATTAATTTATTTGTGAAACGACCTAGGAAACGAAGGAATCAACTTTACAGTCTGATCTTTTCGCGTAAACCTTAACGTTTATTTGTGATAAAAAAAATAAAAACTTTATGCATGTGTGTTCCTAGTGATTTAAAAGTTAATGTTGAAAAAATAAAGTATAAAAAATATTGAAATTATCTTCAAAATTAAAAATTAAAATTTAACTTTGTTGATTGTGGCTAATTAATAAGCTGATGAAGAGCAACGATGAAGCTGTTTACATACATGACAAGCCATGCAGCATGGTGACAGCTTTTCCTAAGCCGAGCCGGCGATTCTACGCACATAAATACCACCGTACGTCTCGCTCGTTCATCAATTTGCGGCTGCCTCCACCTCGATCGATCCATCGCACACTAACTAACAGAGCTAAGATACGACG

>OsGLP9-2

TTGCAATCTTACTCACCTTCTTATATGCTAAATCTGCATATATCCACATATATACTCAGTCAGACAGTCACCCTCTTATATGCCAGTCTGCATATATCCACAAGACAGAAATATTGATGATGGTCAGATGGATAACATGGATCTCAATATAAACAACTGCAATATGCATCACCAACTAAAACTAAGAATTTAACATCTGTTATGTTTCTCCTGAATCCTAACATATAAGCCCTCAAGAGTCAAGAGCACCATTCTAGCAACCCATATTCAGAGCAGTTCTGTTAAATTACAGTGGGCTCTCAAGTTGAATTCTCTTATAGTATCAAACAAAGTTAATTTATACTAGTAATATACATCATTGCATTGTTACATCTGTCTGAACAATGCTCAACACCGAGTGTACCTACCAACGACGTCCATTTCCATTATCAAAGCTTATCCATTGTTTGTCTCAGTTGTAGCATGCCTTTTCCCGTTTGTGGTTTCTACAGTTCACTGATTGCATTTTACCTGTTCATCTCACATGCTTTGAATTTCCACTCTACTACAGTAGCTAAAAAGGGAAACTTCCACCATCCTCACTGCCAAACAGTAGCTAAAAAGGGAAACTTCCACCATCCTCACTGCCAATTGGTCCATGTATTTACCATTATGACAAACAACTTCAAATCTTCTTTTGAAACCGACGGGAGGAGCACTACTGGATATATTAAGGAGGAGCACAGAAGCCTACCAGAAGGCAGTACAGACACAAACAACTTCAAATCAAAACACTGAGGACAAGCACGCAAAAAACAACAAGTCTTGGTGTGTAAGAGTTAATAGACCTGCATTGTTTGATAATTCAGTTGAACTAGAAAGGCAACTGGACCGCACAGCTGAGCTGAGTTATCAATGGAAGATATGTCATTCCTGACGAAGCAGTAGTGAATTGGCAATGAGATATTCTTGCAGTGCAGCTGTAGGTGGGTAGGACTTTGATGCCTTGGGATGACATTTGCTTTCTCCCTTGGTTTCTCATCATGCATCTTCCTTGCTATTATTCATAGTAAACAACCAGGGGAATGCAGAATACGTCCAAGATAACTGATGGAGTTAACAATCTGACTTCCTAATTTAACAAGCTAAGTAGCATGGCATCAGATACGTATTCATTCTCGGAGGCCAGAAATCTAATCTGTTCTGTTCTGTTCTGGACGGGCATAAGTTTTAACAGGAACCTCTAGCTGGCTAGTTTCTTTATCTTGATGCACCGATCTTTCGGTCGTCCAACAACGGAAGATGAATGATTAATTTACGCATAGTATATGTCAATTTCAACCTCGATTTCTCCGGCTTATCGTCAGACGCTTCTTCTGCATAAATAGCCAAATCCTACTGCTCACTGTCTCCATAACACAAGTAGATTGAAGACTAGACCACATCAGTCAAATTAAACTTCGTCTTAACTCATTACTTACTGACAGTTTAGCAGCTGATCGAACGATTAAAATAGATTCA

>OsGLP9-3

TTGAGTGATGCCAATGAGCAAAGGAGTAATGATTTGTTAGGCCTCTGTGCATGTTTGTTTGGTTTCCATGCGCCAAACTATATAGTTGATTTGTGAATTTTTATTAGTACCTGAAACTACTGTTATTAGTATGGCAAGTTTGATCTTGCTCTTTTCCCCTTGCATGTGCAACATATAGCATATTCTCTTTTGGCAATGTAACTCTTAACGTCATCATTGTTGTTTTAGCCTTCTGTGTAATTTATACCTAGATGTTGTACAGCTAGGTAATTTTAATCATCTTGCTCAGTATGGGTAACAAGATTTTATATATTTACCTGCAGATTAATGGGTCCCTGGCTAGGAGGGCTATGAAGGATCTGATGGATAGGGGGCTTATCAGGATGGTGTCGGTTCATTGCAGCCAACAGATTTACACCAGGGCAACGAACACATGATGTTGTCTTATCCATTTTCTAGTCTTATAGACCTGATGTTGCCGTTCTGTTAAGTTTATTCAGTGATACATATGTGGGTGTTCATTTGCCCCAGTAATGTTGAATGTTTTGAGATTTTTGGCACATTGTTAAGGTGATGTTATCTCGGTATCTATCTTGATTATTGCCCAATTAAATTGCACCTTATACGGTGCTACTGCTGTGTCAGTTTGTCTGGTGTTTTCGGTTCCATTATGCCAAGAATTATTTCTGCGTCAACTCAGACGCATTGTGATCTCAGTGGGCTCGGCCGTGTTAAGCCTCTTCCAAAGTCAGCTGAGTTGTGCCTGCCCTGGATCATCAGAAGCTAGTAAACATGTCATTTTACGTCTTGATTTGGTGGAGCATGATAGCGTATTACTAGTTTTCGCTAGTACTGTTATCAGTTTAGAAGCCATTTGTTTGGCGATGAAGCTATTAATAGCTTTTAGCTTGTTGTGTGCTGTTTATCGCGAGATGTTGCACACGAAAATCGTCTTGCAAAGCATCAGGAACCTGGTTTCAAGGGGGCTGACAAGGTAGATGTGAAGTCGATTCGATATTGCATTCTTTGTCTCCATTTTTAGACTTGATGTTGCCATTTTGTAAACTGTGGTACAAATAGATTAGACTAGTTCGACGCTAAACTCTACTTGTGAGATATTTTTATTAAACATCCAGTATTTACTCAAGTGAAGGCTTTGCGATGCCTGGCACATGCATGATCCTTAACTTCCAAGTCAGATTGTATTATGCCAGCTCCATTCTGAAAATAATTGAATGCAGGATATGCAATTTTGATGCTAGATGCATCCAAGGAGCAAGCTGTCAAAAGCCTTGAGTGTTGCCATTTCGATCGCACAAAAGACAGACGGCAGCATTGGTTCCTCCAATTCCTCCTGGCCAAGAGAAAAGCTTCTTATAAATTGGGTTGCTCATCATCTCCATGGCACAAGCACAAGTGCACAACCCAAGCTGCTTATTAGCAAGATCATTTAGCCTCCAAAGCTTAATCAAGATTCAGGCCATCTGACCTGATACAATA

>OsGLP11-1

CATATTGAATATTGATACAGTTAAAAGTGGCCATGCAGTCAGCATCTTCAATTATACTGATGCTCTTTCAGGCACAATAACATCATTACGTCAACCCGTCTAGTGTATAACGCTGTAATGGGATTTGGATGTACATCCTATTATTTGTCCTGGATCACAATTTTTTTACCTAGATAAAGAAAGTAATTTCCCCATCTTTGTGTGGTTAGGTAAATTGTTTTATAGGTCAGTTCATTTCTCAGTTGTCCATCATCTTCTATATGGCTGTACCCATGGGTTTGTGCTGTTATCATTCATGCATCATATATTTTCCCTTGCAATATTGTTTTTAAAATACTTACTATTTCTTGGTTAGTACCTTTAGTCTTGGCTCTCTTTGCTAATGTATCTTCTTAAAATTTTCAGGGCAATTTTCAGACTCTTTACTTCAGCAATGGATTGGCTAAAGAGTAACAAGTACTTCCATATTGTGGTTAAAGGGGTTGAACGTGATGGCTGGAAATTTGTGCTACTTGCTAGATTCTCTCCTCTACCTTCCTACATAATTAACTATGCTCTATCTGCCACCGACGTTGGATTTTTCAAAGATTTTCTACTTCCCACAGTTGTTGGCTGCTTACCGATGATCCTACAGAATGTTTCTATTGTTAGCCTTGCTGGTGCTGCAGTTGCCTCAACCACAGGATCTGAGAAGTCTCGGATATACTCCTACTTGTTTCCAGTACTTGGTATCATGTCTAGCATTCTCATTTCGTGGAGGATTAAGCAATATTCTTCTGCCCTTGTTATTCCTGAAGAGCTTAAAAATTCATCTACTAATGGAAAGGCTAATGTGGATGACAAGGCAGTGTCTGAAAATACCAACTCTGGGGAAACTAGGAAAAGGAGGTGACCATCACAATTTCTTGCATATTACTGTGAGGAGCTACTACGAATTATCAAACATTTAGAGTAGATGCAGATCAAGATTATTCCACATTGGTCAATGACTATAGTAAACTATGTTGTCTGTTTCATGCCATTGTTTTGGGGGCATTTAGCAGATGCGCAGTCAGCTACTGTCTTGTTTCTGCCATCAAGCTTTTCTTTTTCTGACACATGCAGATGTTTCACCTTGCAAACATGGTAGAAGTTCATATGCTGTACATCTAAGTTGGGTAGTTACGGTGGTCTAGGATTTTCTTGTTAAATTATATCTAAGGAAACCGGTTTTGATTTCCTTCCCTACTTGTACTGAACCCATATATGACTTGGTAGAATTCAGTGTTCTAATGGACCAGAATTCAGAACTGTTTCTGCTGTTGCAATGTAGAACAGCTGTATCACACCTTTTTATATATATATACTCTTCTTTAGCCACAATCCAAAGCAAATTATGCAGTGAACTCCAAGAATGTTGCACAGGATACTCAGTCTTCTGACTTTGGTTGCACTCCTGTCTCAACACAGTATTTAACCCATGTTCTGCTTCACTGTATTTTTGTCCAACAATTCCATGAC

>OsGLP12-1

TATATTTTGGAAATTGCTAAAAAAAAACTGTCGCAATTTTTTTTTGGTAAAAGCTTTCAAATATAACTATGGCATAGTTGTAGTATAACTATAATATAATTACACTATAGTTACATTGTAACTACATTGTACTTACAATATGCTTACATTCTAATCTTGATATAACTTAGCTATAAAGTTGCATATTTTTTAATCCTTACAAGTCACATTGTAGTTATAGTATAATTATTGTAGAATTAAATTACAACTATACTACAATTATATTTGAAAGTTTTTTACTTAAAAAAATTGTGGTACTTTTTTTTTATAATCCCTTTTTTTATATACAAAACACACTCCTGGAGACACAGGAAGTATAATATAAGAAGCGCTAAAAAGATAAATATTTCCATATAGGGTATGTTTAGCAAATAGGATATGGGAGGTCGGATTGGGAAATGGAAATAGATGAATGGCTAGGATTAAATAAAGTGTGAAGAATGGATGGCTAAGATGCAATGTTGCCTTTTGGAGGGTGGGATATTATTTCTCAATCCTATTTGCCAAACACAGCCATATTGGAAGCTAGTATTTGTTTTAACTCCTCTCCTAGCTATCCATATGCATCTGGCCAGGAGAATGAAGAGCCGGCGGCCGATGTAATTGATGATGTAATTGATCTGTTGAGCTTTCAACTGAATTAACCAAAGTACCATTAGTTATTAGCTTGAAACTGTCTCGGCAGCTGTAGGCAACAGAGAGCGTGTTGATATATTCAGCAATTAACTGCAATACTGACATTATCAACTTCCCTTTTAATTAGCATGTGTGCTAGCTCTTGACTAATTAATCAGACATTCAGACGTTATATTCCCGTAACACACATGGATGCCTGAATATGCAGAGACGAAAGGGACCAAGTATATTTTACATGGATAGAGAAGACATGGTCAATCTACTCGGTTCTTAGTAATAGTTAACGTACATTCACGGTTATTTTTTTACAACTTATAGGTGTCGTATCAAACTTGTATGTTTGTAAAGGGATATATTTTAATTTAATTTAATTGTCTTATCATTTTTTTAAAATCTTCTTGAGAGATAAAATTCATCTCCCTATCTTTATCATAATATGTTTCTCTGCACCTGATAACAGAGCCAGGCAAGACATAATAAATCTAATGCATGCATCATGCATGACAGGCACTGGAATAATCTAATTAATTAATTAGTCGGCCAACCTAGCTGTTGACTGGGAATTAAGATAGATTGAATCCATCAAATTAAATAGTTTCTTTCCCAGATGCAATCTATTAAGAGTTCAAGATCAGTTCAGACTTTAGAGGAGATGAACCAGTCACAAGCTAGCTCAGCCTTTTGTTATTGACCACTTCAAAATCATCTCATGCTCAGAGGCCTATATATATGGATGCATCATTTCCATGGGCAAAGCATCAACTATAGCTATACAAGAATCATCGACCACTACTTGTACACAAGCAAAAGTTCCATCAACTTAAC

>OsGLP12-2

GCTAAAAAAACTAATCACAATTTCATTTTGGTAAATGCTTTCAAATATAACTATGGTATAGTTGTAGTAACTATAATATAATTACACTATACTTACATTGTAACTACATTGTAATTACAATATACCTACATTCTAATCTCTATATAACTTAGCTGTAAAGTTGCATATTTTTTTAATCTTTACAAGTTACATTGTAGTTATAGTATAATTATTGTAGAATTATAGTACAACTATACTGCAGTTATATTTGAGAGTTTTTCACTTTAAAAAATTGTGGTACCTTTTTTTAGATAATCCCTTTTTTATGCAAAACACACTCCTGAAGATAGAGGAAGGATAATATAACAAGCGCTAAAAAGATAAATATTTCTATATATTGGAAGCTAGTATTTGTTTTAACTCCTCTCCTAGCTATCCATATGCAGCTGGCCAGGGGAATGAAGAGCCGCCGGCCGATGTAATTGATGATGTAATTGATCTGGTGAGCTTTGAACTGAATTAACCAAACTACCAATAGTTATTAGCTTGAAACTGTCTCGGCAGCTGCAGGCAACAGAGAGCGTGTTGATATATTCAGCAATTAACTGCAATACTGACATTATCAACTTCCCTTTTAATTAGCATGGGTGCTAGCTCTTGACTAATTAATCAGACATTCAGACGTTATATTGCCATAACACACATGGATGCCTGAATATGCGGAGACGAAAGGGACCAAGTATACTTTACATGGATAGAGAAGACATGGTCAATCTACTCGTTAACGTACATTCACGGTTATTTTTGTTACAACTTGAATGTCATATTAAACTTGTATTGTATATTTGTAAAGAGATATATTTTAATTTAATTTAATTATCTTATCATTTTTTTAAATCTTCTTGAGAGATAAAAATCCATCTCCCTATCTTTATCATAATATGAGCCTGTTCACTTTGATGCCATTTTCTGATAAAGTTGCTAAAATTTTAACTACGTTTAGTTTGTTGCTAAATTTTTAGTAAGCACATATAAAATCTTGCCAAAATTTAGCAACCTTGTTAAAATTTTAGCAAACATACCAAAATTTAGCAATGCTAAAACTTGGTAAGGTTGAAAATGGTAGTAAAGTGCTCTGTGTTTCTATTCACCTGATAACAAAGACAGCCAAGACATCATCAATCTAATGCATGCATCATGCATGACAGGCACTGGAATAATCTAATTAATTAATTAGTCGGCCAACCTAGCTGTTGACTGGGAATTAAGATAGCTTGAATCCATCAAATTAAATAGTTTCTTTCCCAGATGCAATCATTAAGAGTTCAAGACCAGTTCAGACTTCTGAGGAGATGAACAAGTCACTAGCTAGCTCATCCTTTTCTTATTGACCACTTCAGAATCATTTCATGCTCAGAGGCCTATATATATGGATGCTTCCATGGACAAAAGCATAGACTACAGCTATACAAGAAGCATCGACCACTACTTGTACACAAGCAAAAGTTCCATCAACTTAAC

>OsGLP12-3

TAAACTCATGCCTCTGACACTAATTAAGTTGTAGTAAAAAAAATTAGAAGATTTGGTTTCTGCTCGTATGAAAGAGTACAATAATAAGCAATTTTGATAAAAGTACATATACTACACGTAATTCAATTGGTCATATATCCATCATATCTCTATTATTATAAAAATTGAATATGTTTTTGCCGGTATTTTGTTATATCATCCGTGTATGAGTTGGTTTTTAAGTTCGTTTGCTTTTGAAAATAAATATTCGTATTTGAATTGGTTTTTAAGATCGTTCACTTTTAGAAATACAGAAGAAACCATATAAGAAATCTGTTTAAAAAAAACTCGCATACTAACTTGAGACGATCGGACGTCTAACTGCAGCTCATGATTTTCTATATATATATATATATATATATATATATATATCCAAGCGAACTGCTACAGTGAATTTCATCTTAACTAAATCATATAATAGTAATAAGATTAAAATAAACTTTACCCGTTACAACGCACGAGCAATTTTTCTAGTATATATCAAGTTAACAACTCTGCTCCAGCACATGCTGAAGAGGAAGGAAGAGATCGAGTTCGATCGGGCTAATTAACAGAAAGAAAGGGCATAATAGTTTTACTAATCTCTTTTTACCAGTTAAAACTACTACTATAATGTTTTTAGAACGGATGAATTATAATATAGTGCGCGCGAAAAGGACAAATATTTCCATTATTGGAACTATTTCTTTATCTAACCCTGTTTGGGTATCCAGCTGATCGACCAGGAGAATGACGAGCCGCCGGCCGATGTGATCGATCGATCGATCAGCCGGTGATAGAGCTTTGAACTGAAATAATCAAAACTACAAATTAGTTGAAACTGTCTCAGGGCAACGGGGAGCATGTTGATCGATTCAGCAATTACCGATATCCTTTTTAATTAGCATGCATGTGTGCTAGCTCTTGAAAAATCAGACCTTATTATATTGATGTAACACACATGCATGCTTAATACGCGGAGATCATTGACAAATTAAAAGGGCCATATGGAACAAAGTTACTAAAGGAGTAAAGAGGCCAAGTATACGTACGTTACATAGAGAGAAGACATGGTCATATGTTTATCTGCACCTGATACTGTATAACACAGCCAGGCTTCATCATCAATCTAATGCATGCATGACAGGCACTAAAATAATCTAATTGATTAACTAATCGGTCACCGTGTTGCTGGGAAGACAACTTGAATCCATCCACTAATCCATCAAATTAAATAAGTTCTTGCCCAGATGCAAATCTATCAAGACCGGTTCAGACTTTAGAGCAGATGAACAAGTCGTTAGTTCAGCCTTTTTTTATTTTTCTTATTGACCACTTCAGAATCACCTCATTCTCAGAGGCCTATATATATGGATGCATCCTTTCCTGGCCAAAAACCATCAGCTAGCTGTAGCTCCATATACACAACAAGCTAGCATCGAGTAATACTTCTGCAGAAGCAACAGTTCCATCAACTTAAAC

>OsGLP12-4

AATATTCGGCTCAAACGCCAAAGAGTAAGGGTGCGATCCTGATCATAAACCTCATCCAGTTAGCTGGATAGGAGATCAAGATGGAAATTAAGTCGACTAATTGCAATATCGCGTGGTTATCTTATAGATATCGTGATGATCTCGTGGTAACTTTCTCATTTTTAAAACCTCATGGTTTTCCACGATAACTAAGTACCGTAGTAATTATCATGATATCGCTGAGTAGCAGTAGCCATCATAAAAAGGGTAAGGAAGACGATGGCTGATGCAATCGATCTGGTGAGCTTTGAACTGAATTAATCAAAATTACTAGTAGTTAATTACTCTATTTGTTTCATATTATAAGCCGTTTTGAAATTTTTTTTAATTTAAGTTTGATTAAGTTTATAAAAAATATAGTGACATAACATAAAATAAATATATTATCAAAATATATTCAATGTTAGACTTAATAAAACCAATTTGGTGTTTTAGATGTTGCCAAATTCTTTTTACAAATTTAATTAAACTTTGAAAAATTTAACTAGGAAAACAATCAAAACGAAATGAAACGGAGTAAGCGCTATCCTGAGACTTTCTGATCAGGCAGCTGCAGGCAACAAAGCGTGTTGATTCAGCAATTAACTGCGATACTATCATTATTATCAACTTTACTTTTAATTAGACATTGTCATAACACACATGCATGGATCATTGAAAAATTAATAGGCCATTTGGAACAAATATACGAAAGAGACCCACTTATAATTAATTAAGTTTTATAAAAAAGTAATTTAAAAAGTTCGGAACCCTTCCCACCCAGTAAAAAAACGAAGCAGTGGATTAACTTATTAATTAAATATTAATTAATACGATTTTTTAAAGCAACTCGACCATATAATTTTTTATAAAAAAATACAGTTTAGCACTTGTGAAACGAGAGAAAGATGAAAAAGATCGGCATGCATGACAGGCATGCACTAAAATAATCTAACTAATTACTCGGCCAACCGGTTGCTGGGAACATAACTTGAATCCATCCACTAATCCATCAAATTAAAATATTAAAGAGTTTCTTGCCCAGAAGCAATCTACCAAGACGAGGACTTCAGAGGAGAGATGAACCAGTCGCTATAGATCGATCTTCTAGTTGACCACTTCAGAAACATCTAACGCTCAGAGGCTATAAATATGGCTGCATCCTTTCCATATGGCCAAAGCATCAGATATAGCTCCATACACAAAACAACAATCGACTACTTCTACAGGATCTGTAGATCAAGAGCAAAAGTTCCATCAACTTAAACATGGCCGCCTCCAACTTCTTTCTTCTCACAGCTTTCATTGCTTTGGTCGCTACTCAGGCCATGGCTTCTGATCCTAGTCCTCTTCAGGACTTTTGTGTTGCCGACAAGCACTCGCCAGATATATTATTTTATCACTGATCATGCAATTGCATCAAATTGATTACACATTCATGTTGTACATATACATGTTTCTAGCTTCCGATCTGACCATTGT
